# Limosilactobacillus Regulating Microbial Communities to Overcome the Hydrolysis Bottleneck with Efficient One‐Step Co‐Production of H_2_ and CH_4_

**DOI:** 10.1002/advs.202406119

**Published:** 2024-09-12

**Authors:** Heng Wu, Huaiwen Zhang, Ruixiao Yan, Suqi Li, Xiaohui Guo, Ling Qiu, Yiqing Yao

**Affiliations:** ^1^ College of Mechanical and Electronic Engineering Northwest A&F University Yangling Shaanxi 712100 P. R. China; ^2^ Northwest Research Center of Rural Renewable Energy, Exploitation and Utilization of Ministry of Agriculture Northwest A&F University Yangling Shaanxi 712100 China; ^3^ College of Natural Resources and Environment Northwest A&F University Yangling Shaanxi 712100 P. R. China; ^4^ College of Life Sciences Northwest A&F University Yangling Shaanxi 712100 P. R. China

**Keywords:** CH_4_ production, H_2_ production, hydrolysis enhancement, lactic acid bacteria, microbial mechanism

## Abstract

The efficient co‐production of H_2_ and CH_4_ via anaerobic digestion (AD) requires separate stages, as it cannot yet be achieved in one step. Lactic acid bacteria (LAB) (*Limosilactobacillus*) release H_2_ and acetate by enhancing hydrolysis, potentially increasing CH_4_ production with simultaneous H_2_ accumulation. This study investigated the enhanced effect of one‐step co‐production of H_2_ and CH_4_ in AD by LAB and elucidated its enhancement mechanisms. The results showed that 236.3 times increase in H_2_ production and 7.1 times increase in CH_4_ production are achieved, resulting in profits of 469.39 USD. Model substrates lignocellulosic straw, sodium acetate, and H_2_ confirmes LAB work on the hydrolysis stage and subsequent sustainable volatile fatty acid production during the first 6 days of AD. In this stage, the enrichment of *Limosilactobacillus* carrying *bglB* and *xynB*, the glycolysis pathway, and the high activity of protease, acetate kinase, and [FeFe] hydrogenase, jointly achieved rapid acetate and H_2_ accumulation, driving hydrogenotrophic methanogenesis dominated. From day 7 to 24, with enriched *Methanosarcina*, and increased methenyltetrahydromethanopterin hydrogenase activity, continuously produced acetate led to the mainly acetoclastic methanogenesis shift from hydrogenotrophic methanogenesis. The power generation capacity of LAB‐enhanced AD is 333.33 times that of China's 24,000 m^3^ biogas plant.

## Graphical abstract

This study proposes a novel strategy for one‐step co‐production of hydrogen and methane through the addition of lactic acid bacteria (*Limosilactobacillus*), overcoming the limitations of conventional two‐stage anaerobic digestion processes. Mechanistic analysis confirms the strengthening effect of *Limosilactobacillus* in the hydrolysis phase, and preliminary economic analysis indicates significant potential for the application of this strategy.

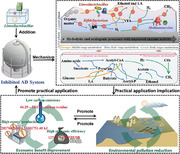

## 1 Introduction

Large‐scale agricultural development has led to a large quantity of livestock manure (LM) produced every year (in excess of 3.8 Mt),^[^ ^1^ ^]^ LM rich in organic matter is a potential energy source. Anaerobic digestion (AD) can use LM to produce energy, that is, hydrogen (H_2_) and methane (CH_4_), which not only avoids the discharge of LM but also obtains clean energy.^[^ ^2^, ^3^ ^]^ Compared with high‐temperature AD (55 °C), lower temperature, such as medium‐temperature AD (35 °C), is a low‐cost option and has therefore attracted widespread attention.^[^ ^4^ ^]^

However, the lower temperatures can lead to higher substrate viscosity, impeding the mass transfer processes.^[^ ^5^ ^]^ This defect can be overcome through stirring measures. More importantly, the enrichment of efficient hydrolytic phyla, such as Firmicutes and Thermotogae, is inhibited in medium‐temperature AD,^[^ ^6^ ^]^ which is accompanied by lower metabolic energy^[^ ^7^ ^]^ and reduced hydrolytic enzyme activity.^[^ ^8^ ^]^ This ultimately results in lower hydrolysis efficiency at medium temperatures,^[^ ^9^ ^]^ thus restricting AD application.

Hydrolysis is the initial step of AD, and its efficiency directly determines the performance of the subsequent acidogenesis step, ultimately impacting the production of H_2_ and CH_4_.^[^ ^10^, ^11^ ^]^ Many physical and chemical techniques have been proposed to improve substrate hydrolytic efficiency and energy recovery, but they are costly and unsustainable.^[^ ^12^, ^13^ ^]^ In the case of biological approaches, the addition of specific microorganisms can reconstruct new microbial communities to drive H_2_ and CH_4_ production pathways, not only promoting the AD process fundamentally but also having the advantage of cleanliness.^[^ ^14^ ^]^

Due to the different characteristics of biological reactions between H_2_ and CH_4_ production, the current approach is a two‐stage AD process for producing H_2_ followed by CH_4_.^[^ ^15^ ^]^ However, the two‐stage AD is intricate and entails high management difficulty. Therefore, simultaneous production of H_2_ and CH_4_ in one stage is practically necessary but undoubtedly challenging.

Lactic acid bacteria (LAB) have the capability of efficient protein and monosaccharide degradation,^[^ ^16^ ^]^ such as *Limosilactobacillus*.^[^ ^17^ ^]^ Its whole genome annotation has confirmed its potential to hydrolyze macromolecular organic matter.^[^ ^18^ ^]^ This LAB can achieve hydrolysis of polysaccharides, lipids, and proteins by secreting enzymes such as glycosyltransferases,^[^ ^19^ ^]^ esterases^[^ ^20^ ^]^, and proteases.^[^ ^21^ ^]^ In theory, LAB addition can enrich hydrolytic genes^[^ ^22^ ^]^ and enhance substrate hydrolysis and glycolysis processes,^[^ ^17^, ^23^ ^]^ which promotes acetate production and H_2_ release. This also provides substrates for acetoclastic and hydrogenotrophic methanogenesis pathways in the AD system, resulting in increased CH_4_ production. In addition, LAB is most suitable for enrichment in a medium‐temperature environment,^[^ ^24^, ^25^ ^]^ making them applicable in this study to overcome the bottleneck of hydrolysis under medium‐temperature conditions. Also, LAB inoculants have been produced on a large scale, which is widely distributed,^[^ ^24^ ^]^ having great application potential. Therefore, AD with LAB addition has the potential for one‐step co‐production of H_2_ and CH_4_, making it a practical and operationally feasible strategy. However, the current understanding of the enhancement effect of LAB on AD remains unknown. In addition, the related mechanisms are not clear. Importantly, the economic benefits are a key indicator for large‐scale practical applications and should not be overlooked.

Therefore, in this work, based on total solid (TS) ratios, LAB was added to the AD system with the co‐production of H_2_ and CH_4_ per unit of volatile solid (VS) in one step. The foci of consideration were: 1) the feasibility of LAB promoting AD system and its advantage over common two‐stage AD, 2) the specific stage of the AD process in which LAB is most effective, 3) the energy generation mechanisms needing elucidation, and 4) importantly, the environmental implications and engineering application potential of the technology.

## 2 Results

### 2.1 Efficient Co‐Production of H_2_ and CH_4_

**Figure 1:**
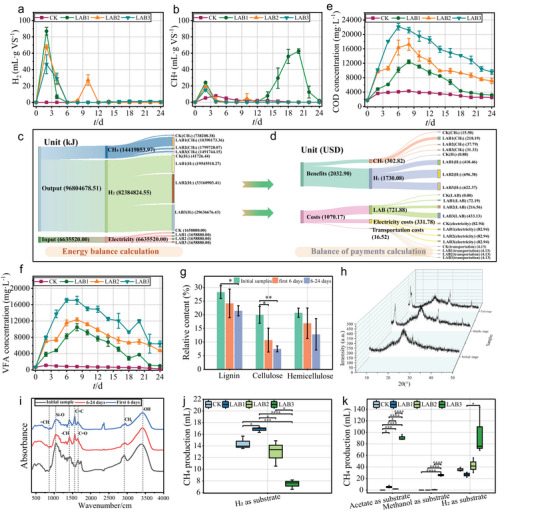
The enhancement effect of LAB addition on AD system and its verification. a) Daily H_2_ production. b) Daily CH_4_ production. c) Energy balance calculation. d) Balance of payments calculation. e) Fluctuations in COD concentration under different LAB inoculation conditions. f) Fluctuations in VFA concentration under different LAB inoculation conditions. g) Lignocellulose content characteristics in the hydrolysis verification experiment. h) X‐ray diffractometer (XRD) characteristics of lignocellulose in the hydrolysis verification test. i) FT–IR characteristics of lignocellulose in the hydrolysis verification test. j) CH_4_ production characteristics during the first 6 days of different pathways in the methanogenic pathways verification test. k) CH_4_ production characteristics from day 7 to 24 of different pathways in the methanogenic pathways verification test. Significance was evaluated by *t*‐test: ****P < 0.0001, ***P < 0.001, **P < 0.01 and *P < 0.05.

LAB was added at ratios of 0.5, 1.5, and 3 g gTS^−1^, corresponding to LAB1, LAB2, and LAB3, respectively, to enhance AD, and compared with the control check (CK). For the co‐production of H_2_ and CH_2_, LAB2 (1.5 g gTS^−1^) achieved the highest H_2_ accumulation (125.20 mL gVS^−1^) (**Figure** **1**a) at conditions of pH 6.23 (Figure S1a, Supporting Information), significantly (P < 0.05, Figures S1b,c, Supporting Information) higher than CK. As shown in Figure 1b, due to continuous significant (P < 0.05, Figures S1d,e, Supporting Information) increased CH_4_ production, LAB1 (0.5 g gTS^−1^) obtained the highest CH_4_ production (196.73 mL gVS^−1^) at conditions of pH 6.74 (Figure S1a, Supporting Information). Importantly, with LAB addition, H_2_ (Figure S1f, Supporting Information) and CH_4_ (Figure S1g, Supporting Information) production from the one‐step AD were higher than two‐stage AD, indicating that sustainable co‐production of H_2_ and CH_4_ via one‐step AD can be achieved by LAB. Also, the use of LAB to enhance AD has overcome the limitations of medium‐temperature conditions, exceeding the performance of AD under high‐temperature conditions (Figures S1h,I, Supporting Information).

After LAB addition, more carbon was used in the AD system (Figure S2, Supporting Information). According to the energy budget (as heat) calculations,^[^ ^26^ ^]^ it confirmed that the net energy returns were enhanced by up to 20.07%, while the CK experienced a loss (Figure 1c). As a key factor in measuring the application potential of the method, the economic benefits were calculated based on the literature^[^ ^27^ ^]^ (Figure 1d). By converting thermal energy to electrical energy with an efficiency of 0.42 (at a retail electricity price of 0.18 USD kWh^−1^), a profit of 469.39 USD was ultimately achieved under LAB1 conditions. The device cost determines the economic return period. Compared with other AD systems, the economic return period of LAB1 has been reduced to 240 days.

In terms of the efficient co‐production of H_2_ and CH_4_, with the increase of LAB addition, the peak value of chemical oxygen demand (COD) significantly (P < 0.05, Figure S3a, Supporting Information) increased from 4286.32 mg L^−1^ in CK to 13,515.79 mg L^−1^ in LAB1 (Figure 1e). The significant (P < 0.05, Figures S3b,c, Supporting Information) increasing electrical conductivity (EC) value also confirmed that hydrolysis was promoted. Efficient hydrolysis led to a rapid increase in acetate concentration in LAB1, reaching 6065.20 mg L^−1^ (Figures S3d–h, Supporting Information), while the maximum concentration in CK was only 859.26 mg L^−1^. This directly resulted in volatile fatty acid (VFA) concentration in LAB1 reaching a peak of 10,468.61 mg L^−1^, which is 9.35 times higher than that of CK (Figure 1f; Figure S3d, Supporting Information). Additionally, the ethanol concentrations in CK and LAB1 decreased from 42.09 and 43.96 mg L^−1^ to 8.97 and 6.31 mg L^−1^, respectively (Figures S3i,j, Supporting Information). Also, the lactic acid concentration in LAB1 reached 1257.02 mg L^−1^, which is 3.37 times greater than that in CK (Figures S3k,l, Supporting Information). The ethanol consumption and lactic acid accumulation confirmed the promotion of acetoclastic methanogenesis through microbial metabolic transformation.

To verify the strengthening effect of hydrolysis in LAB1, 0.5 g gTS^−1^ LAB was added to hydrolyze straw. Waste activated sludge and cow manure were not added to remove the influence of methanogen and organic matter. The hydrolysis resulted in a 43.92% reduction in VS, accompanied by a continuous production of H_2_ with a pH decrease (Figure S4, Supporting Information). The reduced lignocellulose content (Figure 1g) and cellulose crystallinity (Figure 1h) indicated that straw was degraded. Fourier transform infrared spectroscopy (FT–IR) analysis (Figure 1i) showed that the hydrogen bonding, and carboxyl, aldehyde, ketone, and ester groups were reduced, revealing the deconstruction details. More importantly, the methanogenic pathway in LAB1 was determined. Figure 1j shows that only the hydrogenotrophic methanogenesis pathway obtained CH_4_ during the first 6 days, and it had the highest production (16.83 mL). From day 7 to 24 (Figure 1k), the H_2_ and sodium acetate could be used for CH_4_ production, resulting in 5.47 and 26.70 mL respectively, leading to the highest CH_4_ production potential.

### 2.2 Lactic Acid Bacteria Addition Drove the Reconstruction of the Microbial Community

**Figure 2:**
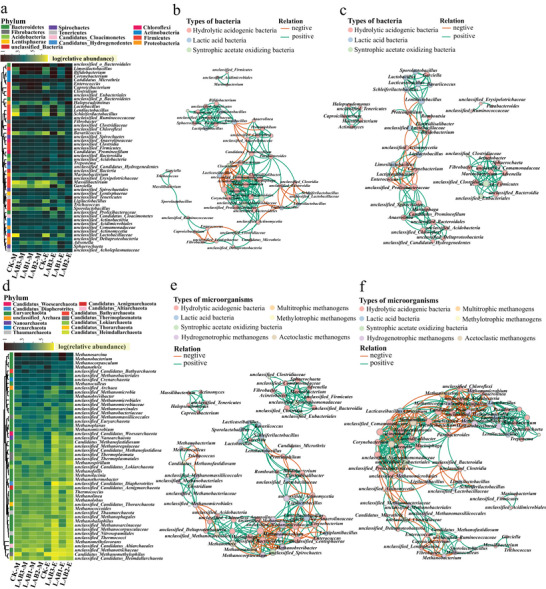
Effects of LAB addition on the microbial community. a) Unrooted phylogenetic tree based on maximum likelihood method, and bacterial relative abundance. b) Bacterial co‐occurrence networks based on *Pearson* correlation during the first 6 days of AD (P < 0.05), where orange lines represent negative correlations and cyan lines represent positive correlations. c) Bacterial co‐occurrence networks based on *Pearson* correlation from day 7 to 24 of AD (P < 0.05), where orange lines represent negative correlations and cyan lines represent positive correlations. d) Unrooted phylogenetic tree based on maximum likelihood method and archaeal relative abundance. e) Archaeal co‐occurrence networks including bacteria and archaea based on *Pearson* correlation during the first 6 days of AD (P < 0.05), where orange lines represent negative correlations and cyan lines represent positive correlations. f) Archaeal co‐occurrence networks including bacteria and archaea based on *Pearson* correlation from day 7 to 24 of AD (P < 0.05), where orange lines represent negative correlations and cyan lines represent positive correlations.

The LAB addition has led to changes in the alpha diversity of bacterial (Table S1, Supporting Information) and archaeal communities (Table S2, Supporting Information). Principal component analysis (Figure S5, Supporting Information) and redundancy analysis (Figure S6, Supporting Information) confirmed that the bacterial and archaeal community was reconstructed due to the LAB addition during the first 6 days, while the two communities were altered by both pH and LAB addition in 6–24 days. To clearly show the interaction among bacteria, the functions of these genera were identified based on genomic data and distinguished as LAB, and hydrolytic and acidogenic bacteria (HAB). As shown in **Figure** **2**a, during the first 6 days, VFA concentrations directly inhibited the enrichment of HAB *unclassified_Bacteroidales* ^[^ ^28^ ^]^ (20.17%) and *unclassified_Bacteroidetes* ^[^ ^29^ ^]^ (2.55%) in CK (Figure S7a, Supporting Information), leading to the dominance of LAB *Limosilactobacillus* ^[^ ^30^ ^]^ (13.20%), and HAB *Enterococcus* ^[^ ^31^ ^]^ (7.30%) and *Clostridium* ^[^ ^32^ ^]^ (2.65%) in LAB1. However, lactic acid and ethanol did not significantly inhibit bacterial enrichment. The co‐occurrence networks were mainly composed of *Limosilactobacillus*, *Ligilactobacillus*,^[^ ^33^ ^]^ *Lacticaseibacillus*,^[^ ^34^ ^]^ and *Clostridium*. The detection of *Clostridium* showed that these genera had a synergistic process of hydrolysis and VFA production (Figure 2b). However, from day 7 to 24, although the abundances of *unclassified_Bacteroidales* and *unclassified_Bacteroidetes* in CK further increased to 31.01% and 3.99%, respectively, the VFA production from hydrolysis remained lower than that in LAB1, which was enriched with *unclassified_Bacteroidales* (9.05%), *Corynebacterium* ^[^ ^35^ ^]^ (0.14%), and *Limosilactobacillus* (0.04%). Changes in environmental factors (e.g., pH) could influence the symbiotic network among microorganisms,^[^ ^36^, ^37^ ^]^ thereby driving the cooperation between *Limosilactobacillus*, *Corynebacterium*, and *Enterococcus* for hydrolysis and VFA production (Figure 2c).

As shown in Figure 2d, during the first 6 days of AD, LAB addition reduced the abundance of *Methanosarcina* ^[^ ^38^ ^]^ to 48.51% in LAB1, which was lower than the 74.71% observed in CK. High VFA concentrations, rather than lactic acid and ethanol, inhibited the enrichment of *Methanosarcina* (Figure S7b, Supporting Information). However, the rapid hydrolysis induced by *Limosilactobacillus* enrichment led to a large amount of H_2_ production, which drove the enrichment of *Methanocorpusculum*,^[^ ^39^ ^]^ ultimately facilitating hydrogenotrophic methanogenesis (Figure 2e). From day 7 to 24, the abundance of *Methanosarcina* increased to 74.66% in LAB1, higher than the 35.65% found in CK, indicating the assembly of a more high‐performance microbial community (Figures S8,S9, Supporting Information). Increased pH led the *Methanosarcina* to cooperate with *unclassified_Bacteroidales* for CH_4_ production (Figure 2f). However, excessive LAB addition in LAB2 and LAB3 resulted in a pH value below 4.0 (Figure S1a, Supporting Information), which suppressed the enrichment of *Methanosarcina*.

### 2.3 Lactic Acid Bacteria Addition Drove the Enrichment of Hydrolytic and Acidogenic Genes

**Figure 3:**
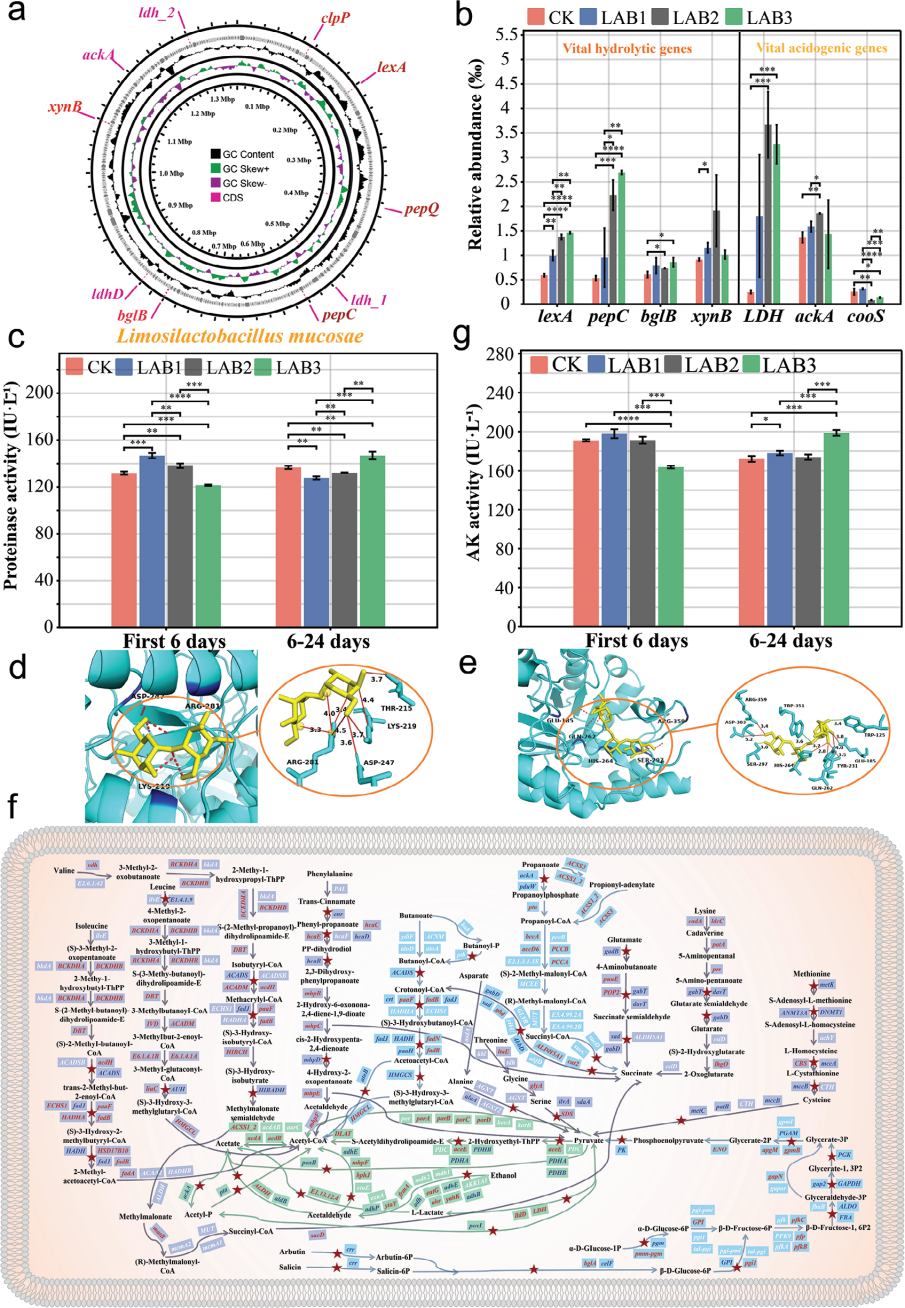
Effects of LAB addition on the enrichment of genes involved in hydrolysis and VFA production. a) Genome circle map of *Limosilactobacillus mucosae*. b) Key high‐abundance enriched genes involved in hydrolysis and VFA production. c) Proteinase activity characteristics in the AD process. d) Optimal molecular docking model of cellulose and *bglB*, as well as the schematic diagram of the interaction force. e) Optimal molecular docking model of hemicellulose and *xynB*, as well as the schematic diagram of the interaction force. The enzyme protein, substrate, amino acid residue, hydrogen bond, and hydrophobic force are labeled in cyan, yellow, blue, red, and green, respectively. f) Hydrolysis and VFA production pathway map reconstructed according to KEGG. The pathway marked by the red pentagram indicates that at least one regulating gene is higher than CK in the LAB system during the first 6 days or subsequent 18 days, while blue indicates that all regulating genes are higher than CK in the LAB system in the whole AD process. Dark blue represents amino acid metabolism, light blue represents VFA metabolism and green represents polysaccharide metabolism. g) AK activity characteristics in the AD process. Significance was evaluated by *t*‐test: ****P < 0.0001, ***P < 0.001, **P < 0.01 and *P < 0.05.

Since microbial metabolic activity is directly regulated by genes, revealing the gene enrichment characteristics is crucial for understanding the hydrolysis enhancement mechanisms. The key genes that promote hydrolysis were further identified in LAB1. The genomic sketch confirmed that *Limosilactobacillus* has key hydrolytic genes and acidogenic genes (**Figure** **3**a). The high abundance of LAB *Limosilactobacillus* led to the enrichment of proteolytic gene *sspA* and *srtA* (P < 0.05, Figure 3b), resulting in a significant increase in the activity of the encoded proteases (P < 0.05, Figure 3c). In addition, the *bglB* and *xynB* genes (P < 0.05, Figure 3b) of *Limosilactobacillus* were responsible for lignocellulose (limiting components) degradation (Figure 3b). The functional enzymes encoded by *bglB* and *xynB* provided molecular docking of cellulose (Table S3, Supporting Information) and hemicellulose (Table S4, Supporting Information), respectively, and the optimal binding models with binding energies of −5.9 and −9.0 kcal mol^−1^ were obtained. These two enzymes can stably bind to cellulose (Figure 3d) and hemicellulose (Figure 3e), respectively, and achieve substrate degradation through hydrogen bonds and hydrophobic interactions.

The vital acidogenic pathways in LAB1 have been reconstructed based on the Kyoto Encyclopedia of Genes and Genomes (KEGG) (Figure 3f), along with their abundance of enrichment (Figure S10, Supporting Information). During the first 6 days, the gene abundances of β‐D‐fructose‐1,6P2 metabolized to acetyl‐CoA were 7.02‰, exceeding that of CK at 6.77‰, ensuring a robust production of acetate. From day 7 to 24, the high‐abundance pathway of succinate metabolism to butyrate stood at 3.53‰, exceeding CK (2.22‰). The conversion of leucine to acetyl‐CoA was also observed, with gene abundances at 2.93‰, exceeding CK (2.47‰), thus sustaining acetate production. Importantly, the enrichment of *alaA*, which regulates the conversion of alanine to produce pyruvate, in LAB1 (0.36‰) persisted throughout the entire AD process, exceeding CK (0.34‰), thereby enhancing the pyruvate‐to‐acetate pathway regulated by *poxB*.

The key genes promoting acetate production were present in LAB1. The enrichment of *Limosilactobacillus* led to a notable increase in the abundance of essential *LDH* genes, as demonstrated in Figure 3b, thereby augmenting the pathway for lactic acid conversion to acetate within the day 7 to 24‐time frame. The heightened presence of the *cooS* (Figure 3b) gene of *Clostridium* was key to facilitating acetate production through homoacetogenesis from day 7 to 24. Also, the *ackA* gene of *Limosilactobacillus* was crucial for regulating acetate production. In the entire AD process, not only was this gene enriched (Figure 3b) but the acetate kinase (AK) activity it encoded was also found to be higher compared to CK (Figure 3g and P < 0.05).

In conclusion, in an AD system containing LAB, hydrolytic genes and acidogenic genes are significantly enriched, and the activity of key enzymes is enhanced, resulting in efficient hydrolysis and VFA production.

### 2.4 Lactic Acid Bacteria Addition Drove the Enrichment of CH_4_ and H_2_‐Producing Genes

**Figure 4:**
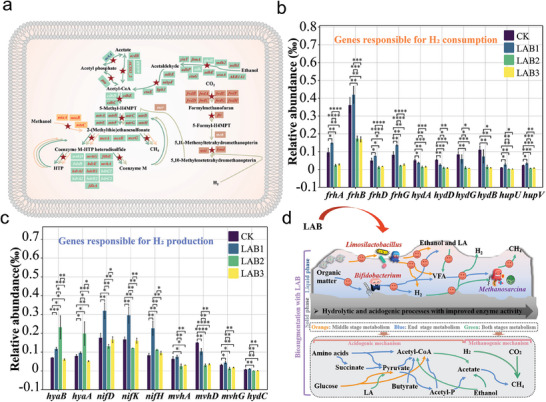
The effect of LAB addition on the enrichment of genes involved in CH_4_ production. a) The main pathway involved in CH_4_ production. The pathway marked by the red pentagram indicates that at least one regulating gene is higher than CK in the LAB system in the entire AD process. b) The abundance of genes responsible for H_2_ consumption in the entire AD process. c) The abundance of genes responsible for H_2_ production in the entire AD process. Significance was evaluated by *t*‐test: ****P < 0.0001, ***P < 0.001, **P < 0.01 and *P < 0.05. d) The microbial metabolic patterns and underlying mechanisms of the LAB‐added AD system.

To further explain the continuous CH_4_ production in LAB1, the reconstructed common methanogenic pathways (**Figure** **4**a) are depicted, along with their enrichment abundance (Figure S11, Supporting Information). During the first 6 days, the gene abundance of hydrogenotrophic methanogenesis was 1.29‰, which was lower than CK at 3.00‰. It was confirmed that the H_2_ released by high‐efficiency hydrolysis was a key factor in achieving high CH_4_ production. From day 7 to 24, the key gene *hmd* regulating hydrogenotrophic methanogenesis pathway was enriched (P < 0.05, Figure S12a, Supporting Information), along with the significantly increased activity of methenyltetrahydromethanopterin hydrogenase (HMD) encoded by *hmd* (P < 0.05, Figure S12b, Supporting Information), confirming that hydrogenotrophic methanogenesis also assisted to CH_4_ production. Importantly, as the most common methanogenic pathway, acetotrophic methanogenesis exhibited a higher abundance of 5.72‰ compared to 4.87‰ in CK throughout the entire AD process, obtaining continuous CH_4_ production. However, in LAB2 and LAB3, the hydrogenotrophic and acetoclastic methanogenesis were both nearly suppressed throughout the entire AD process.

The reason for the accumulation of easily consumed H_2_ in LAB1 needs to be considered. Figure 4b confirmed the enrichment of H_2_‐consuming genes in CK resulted in less H_2_ accumulation. Also, the total abundance of H_2_‐producing genes in the entire AD process was 1.29‰, which was significantly higher than CK at 0.83‰ (Figure 4c), resulting in continuous H_2_ production. The activity of [FeFe] hydrogenase (Hase) encoded by *hydC* was significantly increased (P < 0.05, Figure S12c, Supporting Information), assisting H_2_ production.

In the presence of LAB, as shown in Figure 4d, the abundances of *Limosilactobacillus* and *unclassified Bacteroidales* increased. Also, the abundance of vital hydrolytic genes (*bglB*, *xynB*, *dacC*, and *sspA*) and acidogenic genes (*LDH*, *ackA*, *cooS*), and related encoding enzyme activity (proteases, AK, and Hase) significantly increased. As a result, rapid hydrolysis was achieved, leading to the accumulation of VFA and H_2_. In addition, due to the enrichment of *Methanobacterium* and *Methanoculleus*, high abundance of acetoclastic and hydrogenotrophic methanogenesis pathways, and high activity of HMD, high CH_4_ production was achieved. The low abundance of methanogenic genes and low enzyme activity in LAB2 and LAB3 resulted in low CH_4_ production.

### 2.5 Potential of Lactic Acid Bacteria Mediated Anaerobic Digestion Strategy

#### 2.5.1 Environmental Risk Analysis

**Figure 5:**
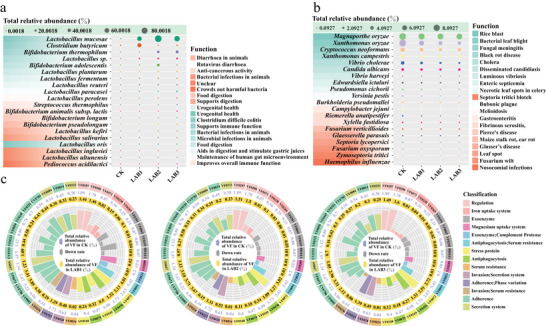
Effects of LAB addition on the pathogenic potential of AD system. a) Composition of probiotics under different AD systems. b) Composition of pathogenic bacteria under different AD systems. c) Virulence factor comparison of CK after LAB addition. The gray rays and font are CK abundance and value, respectively, and the colored rays and fonts are virulence factors abundance and value in LAB, respectively.

For probiotics, LAB1 was dominated by *Lactobacillus mucosae* (57.37%)^[^ ^40^ ^]^ affecting urinary health, while the CK was *Clostridium butyricum* ^[^ ^41^ ^]^ (5.77%) (**Figure** **5**a). Also, the addition of LAB drove the succession of pathogenic bacteria related to biological health risks. As shown in Figure 5b, the *Magnaporthe oryzae* ^[^ ^42^ ^]^ (8.69%) in LAB1 decreased by 22.80% compared to CK. However, the conventional thermal hydrolysis pretreatment‐AD with waste activated sludge as substrates could only remove 13.73% of human pathogenic bacteria at medium‐temperature conditions.^[^ ^43^ ^]^ In addition, as a commonly used method to reduce potential microbial risks, the addition of zero‐valent iron unexpectedly resulted in a 25% increase in the abundance of human pathogenic bacteria.^[^ ^44^ ^]^ Also, the abundance of virulence factors can measure the pathogenic strength of these microorganisms. As shown in Figure 5c, with LAB addition, the main virulence factors (invasion and serum resistance, VF0428) in LAB1 gradually decreased to 4.38% compared to CK, respectively. Therefore, LAB addition is of great significance and value for pathogenic bacteria reduction and biological risk prevention and control.

#### 2.5.2 Comparing the Application Potential in Engineering Scales

**Figure 6:**
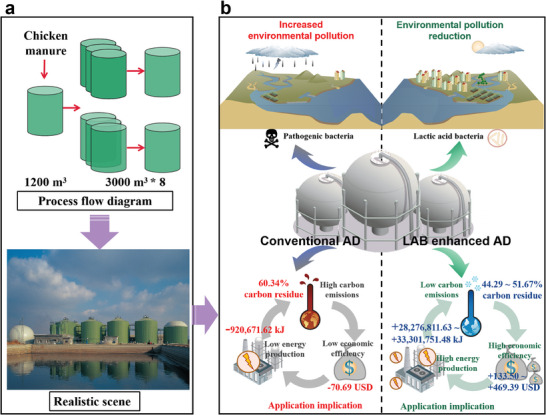
Analysis of the application potential of using LAB to enhance AD. a) Comparing the CH_4_ production potential of the Minhe biogas plant in Shandong, China. b) Concept chart showing the environmental and economic benefits of AD strengthened by LAB.

To evaluate the application potential of LAB addition, the 24000 m^3^ Minhe biogas plant with efficient CH_4_ production at 37 °C was used for comparison (**Figure** **6**a). The Minhe plant generates 22 GWh per year at 300 t d^−1^ treatment efficiency and 10% TS. In contrast, by increasing the treatment efficiency to just 1.5 t d^−1^, comparable annual power generation to the Minhe plant can be achieved. Under the same operation loads, the power generation capacity of LAB‐enhanced AD is 333.33 times that of the Minhe plant. This underscores how the addition of LAB can enhance CH_4_ production and energy conversion efficiency, thereby boosting overall plant operational effectiveness. From the conceptual diagram in Figure 6b, it can be observed that the use of LAB to enhance AD not only reduces pollutants but also effectively reduces the spread of pathogens. Additionally, it improves carbon use, production capacity, and economic benefits, providing a safer and more efficient novel strategy.

## 3 Discussion

The medium‐temperature AD is a widely adopted model in global biogas plants. However, its efficiency limits the potential for generating more energy through AD and makes it nearly impossible to produce H_2_. In contrast, high‐temperature AD enhances energy production but incurs higher costs, conflicting with the sustainable plans of biogas plants. To promote the further application of medium‐temperature AD, measures must be taken to strengthen its efficiency. From a sustainability perspective, adding microbial agents as a biological method is cleaner and more cost‐effective, making it a focus of interest for countries worldwide in the future. In this study, LM and waste activated sludge was used as substrates rich in antibiotics, antibiotic‐resistant genes, and chlorine disinfectants, requiring microbial agents with high stress resistance. Also, considering practical application feasibility, the selected microbial agents should be easy to obtain. Additionally, the microbial growth environment must align with medium‐temperature AD conditions. Therefore, utilizing low‐cost, large‐scale production LAB (*Limosilactobacillus*) to reinforce the hydrolysis stage is an important innovative strategy, ultimately enabling the co‐production of H_2_ and CH_4_. This study has successfully overcome two major technological bottlenecks: first, achieving simultaneous production of H_2_ and CH_4_, thus breaking through the limitations of standard two‐stage AD (Figures S1f,g, Supporting Information); second, exceeding the medium‐temperature constraints and producing more H_2_ and CH_4_ than high‐temperature AD (Figures S1h,I, Supporting Information). This confirms the feasibility and advancement of the LAB‐enhanced AD strategy.

After the addition of LAB, the AD system delivered peak H_2_ production during the first 6 days, followed by peak CH_4_ production from 7 to 24 days. During the first 6 days, the dominant genus *Limosilactobacillus*, having hydrolytic genes *bglB* and *xynB*, replaced the resident bacteria (Figure 2b; Figure S7a, Supporting Information) and degraded the limiting substrate through interactions. Simultaneously, glycolysis is enhanced and *ackA* is enriched, especially with a notable upregulation of the H_2_‐regulating gene *hydC*, leading to the accumulation of substantial quantities of VFA and H_2_. This inhibited *Methanosarcina* and drove *Methanocorpusculum* to produce a small quantity of CH_4_ through hydrogenotrophic methanogenesis (Figure 2d; Figure S7b, Supporting Information). In summary, efficient hydrolysis accelerated H_2_ release while inhibiting H_2_‐consuming pathways, leading to its accumulation. This finding makes low‐cost, high‐purity H_2_ production a possibility.

From day 7 to 24, *Limosilactobacillus* drove the resident bacterial community to reconstruct predominantly around *unclassified_Bacteroidales* (Figure 2d; Figure S7a, Supporting Information), leading the main acidogenic pathways to shift to succinate and leucine metabolism, which continuously enabled the highly abundant and active *ackA* to co‐produce acetate while releasing small quantities of H_2_. At this point, VFA consumption promoted the gradual enrichment of *Methanosarcina* (Figure 2d; Figure S7b, Supporting Information), ultimately producing large quantities of CH_4_ through both acetoclastic and hydrogenotrophic methanogenesis. Therefore, the key reason that enhanced hydrolysis promotes CH_4_ production lies in the release of a significant number of intermediate products, which directly drive the enrichment and succession of the methanogenesis pathways.

Economic feasibility is a critical factor that must be evaluated for the practical application of new technology. After the addition of LAB, carbon residues were reduced, which resulted in greater thermal energy and profits (469.39 USD), thus overcoming the low economic efficiency of standard medium‐temperature AD. Compared to the 24000 m^3^ Minhe biogas plant in Shandong Province, China with an annual power generation is 22 GWh, CH_4_ production can be increased by 333.33 times. In conclusion, LAB enhances the efficiency of the AD process, accompanied by substantial profits, indicating a broad application prospect. To our knowledge, this is the first evaluation of the use of LAB to achieve the efficient co‐production of H_2_ and CH_4_ in one‐stage AD, and elucidation of the underlying microbial mechanism. It is important to highlight that this work has demonstrated that this approach is highly applicable to widespread adoption holds significant economic value, and is particularly valuable for factories globally engaged in the treatment of agricultural waste for resource utilization purposes.

## 4 Experimental Section

### Sources of Waste Activated Sludge, Cow Manure, and Lactic Acid Bacteria

The waste activated sludge was obtained from a wastewater treatment plant in Yangling District, Shaanxi Province, China. Tap water was added to adjust total solid (TS) and volatile solid (VS). The cow manure was taken from the Animal Science Experiment Station of Northwest A&F University (Xianyang, China). The lactic acid bacteria (LAB) (*Limosilactobacillus*) mixed microbial inoculum was purchased from Chuan Xiu Co., Ltd (Beijing, China). **Table** **1** shows their TS, VS, total carbon (TC), and total nitrogen (TN).

**Table 1:** Chemical properties of LAB inoculum and substrates used to evaluate LAB‐amended anaerobic digestion systems.

	TS	VS	TC	TN	pH	NH_4_^+^‐N[mg L^−1^]	COD [mg L^−1^]
LAB	93.18%	91.44%	41.29%	0.24%	‐	‐	‐
Waste activated sludge	3.65%	2.28%	27.44%	3.91%	8.31	768.61	792.31
Cow manure	18.10%	13.84%	37.77%	2.35%	7.67	318.52	2859.62

### Experimental Procedure

Based on their VS contents, waste activated sludge, and cow manure were mixed in a 1:4 ratio to achieve a final TS of ≈6% for the substrate, with a total volume of 350 mL. This substrate mixture was then placed in 500 mL fermenters for batch AD. LAB was inoculated to separate fermenters at 0, 0.5, 1.5, and 3 g gTS^−1^ (designated CK, LAB1, LAB2, and LAB3, respectively). The fermenters were then placed in a 1 m^3^ incubator (SPM‐1008, Ningbo, China) for batch AD at a constant temperature of 35 °C. The AD was run for 24 days. Every 2 days, 1 mL samples of the mixed solution from the fermenters were collected and diluted for measurement of COD and VFA concentration, and to determine CH_4_, H_2_, CO_2_, and electrical conductivity (EC). The pH of fermenters was adjusted to ≈7.0 using 6 mol L^−1^ NaOH and HCl.

This experiment had three replicates of each treatment, with microbial samples taken from each replicate to test the reproducibility of the fermenters. Biosamples for microbiological analysis taken from fermenters with LAB addition ratios of 0, 0.5, 1.5, and 3 g gTS^−1^ was named CK, LAB1, LAB2, and LAB3, respectively. The samples in the first 6 days (AD middle stage) were labeled with the suffix M, such as CK‐M. Similarly, the samples in the subsequent 6–24 days (AD end stage) can be named CK‐E. This naming convention can be applied to other samples as well.

To verify the potential and advantages of co‐production of H_2_ and CH_4_ through the addition of LAB to the one‐step AD system, a comparative two‐stage AD system was also evaluated. For this, the substance was again cow manure and waste activated sludge, with a TS of 6% and AD run at medium temperature. Likewise, four treatments were evaluated with LAB additions 0, 0.5, 1.5, and 3 g gTS^−1^. These sets were named two‐stage CK (TCK), two‐stage LAB1 (TLAB1), two‐stage LAB2 (TLAB2), and two‐stage LAB3 (TLAB3), respectively. The AD was run for 24 days, with the first 12 days used to adjust the pH to ≈5.2 to establish a VFA and H_2_ production system, followed by the next 12 days where the pH was adjusted to ≈7.2 to establish a CH_4_ production system.^[^ ^45^ ^]^ In addition, to verify whether LAB‐enhanced AD overcame the constraints of medium temperatures, AD was run in a set of fermenters under high‐temperature conditions. Apart from the temperature, all other parameters were consistent with those of medium‐temperature AD.

### Hydrolysis Verification Test

To assess the enhancement effect of LAB in the hydrolysis process, the restrictive component lignocellulose within the substrate was assessed. LAB was directly introduced into the fermenters with straw as the substrate. The pH and H_2_ production during the AD process was monitored, while also comparing the lignocellulose content, Fourier transform infrared spectroscopy characteristics, and X‐ray diffractometer characteristics before and after hydrolysis. The experiment used a total of three fermenters, each containing ≈35 g of straw (with a lignocellulose content similar to that of a one‐step AD experiment). Tap water was added to a constant volume of 350 mL. LAB was added at a ratio of 0.5 g gTS^−1^.

### Methanogenic Pathways Verification Test

Sodium acetate, H_2_, and methanol were used as model substrates to evaluate the effect of LAB on CH_4_ production.^[^ ^46^, ^47^ ^]^

### Methanogenic Pathways Verification Test—Acetoclastic Methanogenesis Test

A total of 30 mL of the mixture in these fermenters under each condition was taken as the inoculum, and then they were diluted tenfold. Sodium acetate was used as a methanogenic substrate (5 g L^−1^) in the diluted fermenters. The pH was maintained at ≈7.0, and cultured at a medium temperature. CH_4_ production was detected after 2 days.

### Methanogenic Pathways Verification Test—Hydrogenotrophic Methanogenesis Test

Mixed gas (40% H_2_, 50% N_2,_ and 10% CO_2_) was used instead of sodium acetate, and the experimental process was consistent with the acetoclastic methanogenesis test.

### Methanogenic Pathways Verification Test—Methylotrophic Methanogenesis Test

Methanol (5 g L^−1^) was used instead of sodium acetate, and the experimental process was consistent with the acetoclastic methanogenesis test.

### Assessment of Enzyme Activity

The activities of acetate production, H_2_ production, and hydrolysis were assessed using acetate kinase (AK),^[^ ^48^, ^49^ ^]^ [FeFe] hydrogenase (Hase)^[^ ^50^ ^]^ and protease,^[^ ^51^ ^]^ respectively. The activity of hydrogenotrophic methanogenesis was assessed using methenyltetrahydromethanopterin hydrogenase (HMD).^[^ ^52^ ^]^ All enzyme activity was measured during the first 6 days and the subsequent 18 days of the AD process.

### DNA Extraction, Library Construction, Shotgun Sequencing and Sequence Quality Control, and Genome Assembly

Genomic DNA was extracted from biosamples using the Mag‐Bind Soil DNA Kit (Omega Bio‐tek, Norcross, GA, USA) as per the manufacturer's instructions. The DNA concentration and purity were measured with TBS‐380 (Turner Biosystems, Sunnyvale, CA. USA) and NanoDrop2000 (Bioo Scientific, Austin, TX, USA). Quality assessment was done on a 1% agarose gel. The DNA was fragmented to an average length of 400 bp using Covaris M220 (Gene Company Limited, HK, China) for paired‐end library preparation. The library was constructed with NEXTFLEX Rapid DNA‐Seq (Bioo Scientific, Austin, TX, USA), and paired‐end sequencing was performed on an Illumina NovaSeq at Majorbio Bio‐Pharm Technology Co., Ltd. (Shanghai, China) using the NovaSeq 6000 S4 Reagent Kit v1.5 (300 cycles) following the manufacturer's instructions. All the measurements were made in triplicate. The paired‐end Illumina reads were trimmed of adaptors and low‐quality reads (length <50 bp or with a quality value <20 or having N bases) were removed by fastp (version 0.20.0). Metagenomics data were assembled into contigs (length ≥ 300 bp) using MEGAHIT.^[^ ^53^ ^]^

### Gene Prediction, Taxonomy, and Functional Annotation

Open reading frames (ORFs) in each assembled contig were predicted using Prodigal^[^ ^54^ ^]^/MetaGene^[^ ^55^ ^]^ (http://metagene.cb.k.u‐tokyo.ac.jp). ORFs predicted to be ≥100 bp in length were extracted and then translated into amino acid sequences utilizing the NCBI translation table (http://www.ncbi.nlm.nih.gov/Taxonomy/taxonomyhome.html/index.cgi?chapter=tgencodes#SG1). A non‐redundant gene catalog was compiled using CD‐HIT^[^ ^56^ ^]^ (http://www.bioinformatics.org/cd‐hit, version 4.6.1) with a 90% sequence identity and 90% coverage. SOAPaligner^[^ ^57^ ^]^ was used to compare high‐quality readings with non‐redundant gene catalogs to calculate gene abundance with 95% identity (http://soap.genomics.org.cn, version 2.21). Diamond^[^ ^58^ ^]^ (http://www.diamondsearch.org/index.php, version 0.8.35) was used to compare the representative sequences in the non‐redundant gene catalog with the NR database, and the e‐value cutoff value of 1e^−5^ was used for classification annotation. Diamond^[^ ^58^ ^]^ was used to perform the Kyoto Encyclopedia of Genes and Genomes (http://www.genome.jp/keeg) annotation (http://www.diamondsearch.org/index.php, version 0.8.35), with an e‐value cutoff value of 1e^−5^. The carbohydrate‐active enzyme annotation of the CAZy database (http://www.cazy.org) was performed using hmmscan (http://hmmer.janelia.org/search/hmmscan), and the e‐value was truncated to 1e^−5^. Diamond^[^ ^58^ ^]^ (http://www.diamondsearch.org/index.php, version 0.8.35) was used to annotate the virulence factors of the VFDB database (http://www.mgc.ac.cn/VFs), and the e‐value was truncated to 1e^−5^.

### Molecular Docking

The molecular docking steps were as follows^[^ ^59^ ^]^: According to the shotgun sequencing data, the *bglB* and *xynB* were selected to construct the protease by SWISS‐MODEL (https://swissmodel.expasy.org/interactive) homology modeling. Molecular docking was performed using Auto Dock Tools (https://autodock.scripps.edu), which are used to remove excess solvent molecules and remove any unbound organic contaminants from the selected proteins. The molecular docking of receptors and ligands was done with Vina software, and the docking results were visualized using Pymol software (https://pymol.org/2).

### Analytical Methods

TC and TN were measured by the elemental analyzer (Elementar vario MACRO cube, Langenselbold, Germany). TS and VS were measured by the weight loss method. pH was measured with a pH meter (FE28, CH). The activity of protease, HMD, AK, and Hase were analyzed by assay kits purchased from Shanghai Enzyme‐linked Biotechnology Co., Ltd (Shanghai, China) according to the instructions. EC was measured by a conductivity meter (Youke conductivity DDS‐11A, Shanghai, China). The lignin, cellulose, and hemicellulose were measured according to Van Soest washing fiber analysis method. Crystallinity, expressed as crystallinity index (*CrI*), was determined using a powder XRD (Bruker D8 Advance A25, Germany) with Cu radiation at 40 kV and 40 mA. Samples were scanned in the 2θ range of 5 to 50°. The *CrI* was calculated as [(*I_002_*‐*I_am_*)/*I_002_*]/100, where *I_002_* is the maximum crystalline diffraction intensity of cellulose I at 22° to 23° (for cellulose II, 2θ is 18° to 22°) and *I_am_* is the crystalline diffraction intensity of cellulose I at 2θ of 18° to 19° (for cellulose II, 2θ is 13° to 15°). FT–IR (Vertex70, Germany) was used to investigate organic functional groups and structural changes in fermenters. Spectra were recorded between 400 and 4000 cm^−1^. The methods COD, VFA, H_2_ and CH_4_ determination, and carbon balance, energy balance, and economic and metagenomic binning analyses are given in supplementary materials.

α‐Diversity indices were computed using Mothur v1.30.1, including observed Shannon, Shannoneven, Chao, and Coverage.^[^ ^60^ ^]^ The linear discriminant analysis effect size^[^ ^61^ ^]^ (http://huttenhower.sph.harvard.edu/LEfSe) was performed to identify significantly enriched bacteria and archaea across different samples. The similarity among the microbial communities in different samples was determined by principal component analysis using R package stats. The redundancy analysis was performed using R package vegan (v2.5‐3) to investigate the effect of environmental factors on microbial communities. The abundance data for bacteria and archaea represent the averages from three measurements. Based on the abundance data, co‐occurrence networks were constructed to explore interactions among genera using Pearson's correlation to assess the relationships among bacteria and between bacteria and archaea.^[^ ^62^ ^]^ Significant symbiotic relationships with correlation coefficients with absolute values of >0.5 (P < 0.05) were identified.

### Statistical Analysis

All samples were assayed in triplicate, and the standard deviation analysis and significance testing (*t*‐test) were conducted using Origin 2021.

Significance was evaluated by *t*‐test: ****P < 0.0001, ***P < 0.001, **P < 0.01 and *P < 0.05.

## Conflict of Interest

The authors declare no conflict of interest.

## Author Contributions

H.W. did conceptualization and visualization, performed methodology, formal analysis, investigation, and has written the original draft. H.Z. did formal analysis, and visualization. S.L. performed methodology, and project administration. R.Y. did supervision. X.G. and L.Q. did visualization. Y.Y. did resources, conceptualization, supervision, and project administration.

## Supporting information



Supporting Information

## Data Availability

The data that support the findings of this study are available from the corresponding author upon reasonable request.
