# Supplementary material for: Limosilactobacillus Regulating Microbial Communities to Overcome the Hydrolysis Bottleneck with Efficient One‐Step Co‐Production of H2 and CH4
Source: Adv Sci (Weinh). 2024 Sep 12;11(43):2406119. doi: 10.1002/advs.202406119 (PMC11578306; doi:10.1002/advs.202406119)
Supplement: Supplementary file 1 — Supporting Information [file ADVS-11-2406119-s001.docx]

**Limosilactobacillus Regulating Microbial Communities to Overcome the Hydrolysis Bottleneck with Efficient One-Step Co-Production of H_2_ and CH_4_**

**Heng Wu^1^, Huaiwen Zhang^1^, Ruixiao Yan^2^, Suqi Li^3^,** **Xiaohui Guo^1^, Ling Qiu^1^, Yiqing Yao^1^***

*^1^ College of Mechanical and Electronic Engineering, Northwest A&F University, Yangling, Shaanxi 712100, PR China;*

*^2^ College of Natural Resources and Environment, Northwest A&F University, Yangling, Shaanxi 712100, PR China;*

*^3^ College of Life Sciences, Northwest A&F University, Yangling, Shaanxi 712100, PR China.*

**About the author:**

*Corresponding author.

E-mail addresses: [dzhtyao@nwafu.edu.cn](mailto:dzhtyao@nwafu.edu.cn) (Y. Yao)

Physical mailing address: 22^#^ Xinong road, Yangling District, Shaanxi 712100, PR China

**Supplementary Materials and Methods**

**Carbon balance calculation (g)**: The calculation of carbon balance theory is carried out in a relatively ideal state, without considering the loss of trace carbon caused by manual operation during the experiment. It is believed that the total carbon in the AD system mainly comes from gas (CH_4_, CO_2_), liquid (volatile fatty acid (VFA), ethanol, lactic acid), and residual solid (residual carbon). The mass of carbon in CH_4_ and CO_2_ is calculated by the density at 25 °C ideal state.

Carbon balance equation：*m*_CM_ + *m*_WAS_ + *m*_LAB_ = *m*_CH4_ + *m*_CO2_ + *m*_VFA_ + *m*_LA_+ m_ethanol_ + *m*_residual solid_ + *m*_microbial consumption_  (1)

where: *m*_CM_, *m*_WAS_, *m*_LAB_ represent the quality of carbon in cow manure (CM), waste activated sludge (WAS) and lactic acid bacteria (LAB) respectively, and their sum represents the initial total carbon quality of AD system (g). The *m*_CH4_, *m*_CO2_, *m*_VFA_, *m*_LA_, *m*_ethanol_, *m*_residual solid_, and *m*_microbial consumption_ represent the carbon mass used by microorganisms in CH_4_, CO_2_, VFA, lactic acid, ethanol, AD remaining solids, and microbial consumption, respectively (g).

The calculation of carbon mass in solid:

*m*_CM_=TC × *m*_existing_ (2)

where: TC represents the measured total carbon content in CM (%), and m_existing_ represents the mass of CM existing in the experiment (g). According to this formula, *m*_WAS_, *m*_LAB_, and *m*_residual solid_ can be calculated.

The calculation of carbon mass in gas and liquid:

*m*_CH4_=*V*×*ρ*× *η* (3)

where: *V* represents the volume of CH_4_ (m^3^), *ρ* represents the density of CH_4_ at standard atmospheric pressure (g/m^3^), and *η* represents the relative content of carbon in CH_4_. According to this formula, *m*_CH4_, *m*_CO2_, *m*_VFA_, *m*_LA_, and m_ethanol_ can be calculated. When calculating the carbon mass in the liquid, only the liquid concentration is needed instead of the gas density.

**Energy balance calculation (in heat, kJ)**: The energy output mainly comes from the combustion heat of H_2_ and CH_4_. The energy input mainly comes from the electric energy consumed by the incubator. Considering the huge volume of the incubator, in order to evaluate the energy production efficiency of this work as accurately as possible, the actual production of H_2_ and CH_4_ will be expanded by 250 times.

Energy balance equation：

*E*_output_-*E*_input_=*E*_production_  (4)

*E*_output_=*E*_H2_+*E*_CH4_  (5)

where: *E*_H2_ and *E*_CH4_ represent the heat energy of H_2_ conversion and CH_4_ conversion, respectively, and the sum of the two is the energy output (*E*_output_) of the AD system (kJ). *E*_input_ is the electrical energy consumed by the incubator. *E*_production_ is net capacity (kJ).

*E*_H2_=*V*×*K*_H2_ (6)

*E*_CH4_=*V*×*K*_CH4_  (7)

where: *V* is the volume of H_2_ or CH_4_ (m^3^), *K*_H2_ and *K*_CH4_ are the energy conversion coefficients of the two, which are 143 MJ/m^3^ and 37 MJ/m^3^, respectively [1].

**Economic benefit calculation (USD)**: The efficiency of converting thermal energy into electrical energy is calculated according to 0.42 [2]. The cost of LAB was calculated according to 27.5 USD/kg, and the electricity cost was calculated according to 0.18 USD/kWh.

*Y*_output_-*Y*_input_=*Y*_profit_ (8)

*Y*_output_=*Y*_H2_+*Y*_CH4_ (9)

*Y*_input_=*Y*_Electricity_+*Y*_LAB costs_+*Y*_installation cost_+*Y*_transportation cost_ (10)

*Y*_Electricity_ and *Y*_LAB_ costs represent the cost of electricity and LAB purchase, respectively, while *Y*_H2_ and *Y*_CH4_ represent the benefits of H_2_ and CH_4_ production capacity, respectively.

*Y*_H2_=0.42×*E’*_H2_×0.18 (11)

*Y*_CH4_=0.42×*E’*_CH4_×0.18 (12)

*Y*_LAB costs_=*m*_LAB_×27.5 (13)

where: *E’*_H2_ and *E’*_CH4_ can be calculated based on equations (6)-(7), and converted into electrical energy. The *m*_LAB_ is the mass consumed by LAB (g)*. Y*_Electricity_is about 82.94 USD. *Y*_installation cost_ includes the cost of buying incubators, the cost of buying gas collection bags, and the cost of buying fermenters, totaling 4,640.63 USD. *Y*_transportation cost_ represents the transportation cost of AD raw materials. Due to the short distance, the transportation cost is only about 4.13 USD.

*t*×*Y*_profit_**≥***Y*_installation cost_ (14)

Where: *t* represents the economic return cycle (d).

**The method for determining COD is as follows**: A pre-made COD reagent kit purchased from Titrc Technology Co., Ltd. in China was used. Reagent A in this kit primarily consists of silver sulfate, mercury sulfate, and potassium dichromate, while Reagent B serves as a masking agent to eliminate the influence of chloride ions. The sample to be tested is added to a digestion tube that can withstand high temperatures of 165 °C and placed in a digestion instrument (Thermo Orion COD165, USA) for 30 minutes. After cooling, it is then transferred to a UV-visible spectrophotometer (UV2310II, China) to measure the absorbance. Standard curve drawing method : The potassium hydrogen phthalate solution was used as the standard solution, and the reagent was added for determination after gradient dilution, and then the standard curve was drawn.

**The method for determining VFA is as follows**: The concentrations of VFA (Acetate, propionate, butyrate, isobutyrate, valerate, isovalerate) were determined using a Shimadzu GC-2014C gas chromatograph (Shimadzu Corporation, Kyoto, Japan) equipped with a DB-FFAP capillary column (Agilent Technologies, Wilmington, DE, USA) and a flame ionization detector. After diluting the samples for analysis, the supernatant was treated with metaphosphoric acid for pretreatment, followed by the addition of crotonic acid as an internal standard for measurement. The analytical procedure included an initial temperature of 50°C, held for 3 minutes, then increased to 130°C at a rate of 10°C per minute, followed by a rise to 170°C at 5°C per minute, and finally increased to 220°C at 15°C per minute, maintaining this temperature for 3 minutes. Since the VFA determination was conducted using the internal standard method, a standard sample was prepared by directly mixing acetate, propionate, butyrate, isobutyrate, valerate, and isovalerate purchased from Maiklin Biotech Co., Ltd. in China, with crotonic acid added as the internal standard. After gas chromatography analysis, the retention times for each acid were ultimately obtained.

**The method for determining gas is as follows**: The key gases in biogas (CH_4_, CO_2_, and H_2_) were measured using a Shimadzu GC-2014C gas chromatograph (Shimadzu Corporation, Kyoto, Japan) equipped with a Shimadzu MS-13X packed column. The temperature program was set to include a column temperature of 80°C, a DTCD1 temperature of 150°C, and a DINJ temperature of 100°C. After the temperature ramp was completed, the biogas was directly analyzed, allowing the retention times for the different gas components to be obtained. The standard gas used was a synthetic biogas mixture purchased from Shaanxi Maichi Trading Co., Ltd.

Metagenomic binning and taxonomy annotation of bins: Binning analysis was performed using metagenomic sequencing data to identify key LAB strains and their key genes. Contigs with a length ≥ 1000 bp were selected as the final assembling result, and then the contigs were used for further binning to retrieve metagenome-assembled genomes (MAGs). Binning was performed using MetaBAT [3] (version 2.12.1). The results generated by the above binning tools were integrated using DAS_tools [4] (version 1.1.0) to obtain an optimized, non-redundant set of bins as the final binning result. Then, the contamination of each bin is assessed by examining the GC content, coverage, and tetranucleotide frequency (TNF) of the contigs within each bin. Contigs with better quality are selected based on the assessment results, and contaminated sequences are manually removed to correct the bins. The completeness, contamination, and strain heterogeneity of the bins were estimated by CheckM [5], and the bins with more than 50% completeness were kept for downstream analysis. Then, the taxonomy of the recovered genomes (bins) was determined using a set of 120 universal single-copy proteins based on the Genome Taxonomy Database (GTDB) using GTDB-Tk [6]. The genomic circle diagram of *Limosilactobacillus* was drawn using CGview (https://paulstothard.github.io/cgview/).

**Supplementary Result and Discussion**

**
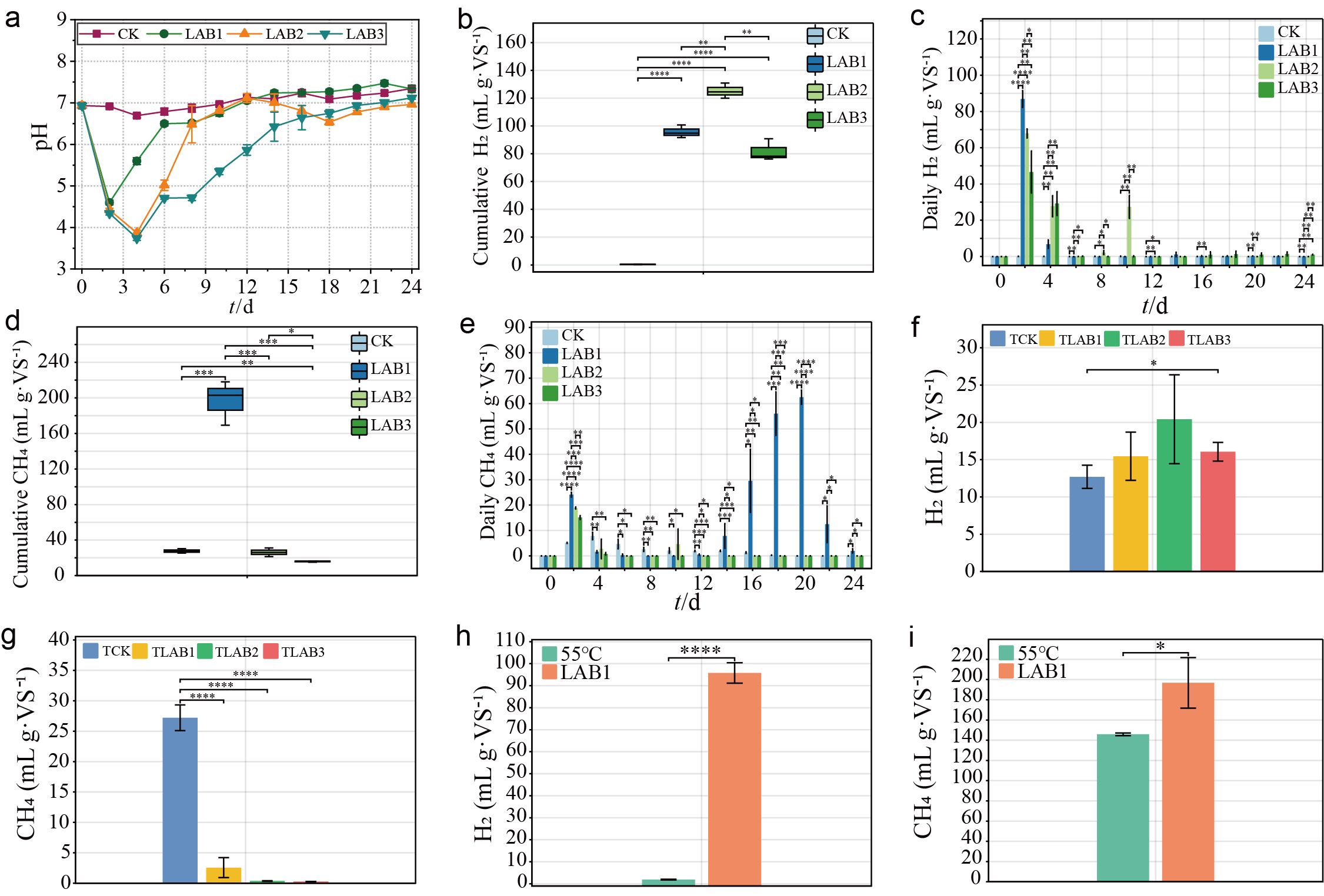
**

**Figure S1**. The pH changes under different LAB inoculation conditions (a). Visualization of significant tests for cumulative H_2_ production (b), visualization of significant tests for daily H_2_ production (c), visualization of significant tests for cumulative CH_4_ production (d), visualization of significant tests for daily CH_4_ production (e). The cumulative H_2_ production (f) and CH_4_ production (g) of two-stage AD under different LAB addition conditions. The cumulative H_2_ production (h) and CH_4_ production (i) of AD under 55℃ conditions. Significance was evaluated by *t*-test: **** P < 0.0001, *** P < 0.001, ** P < 0.01 and * P < 0.05.


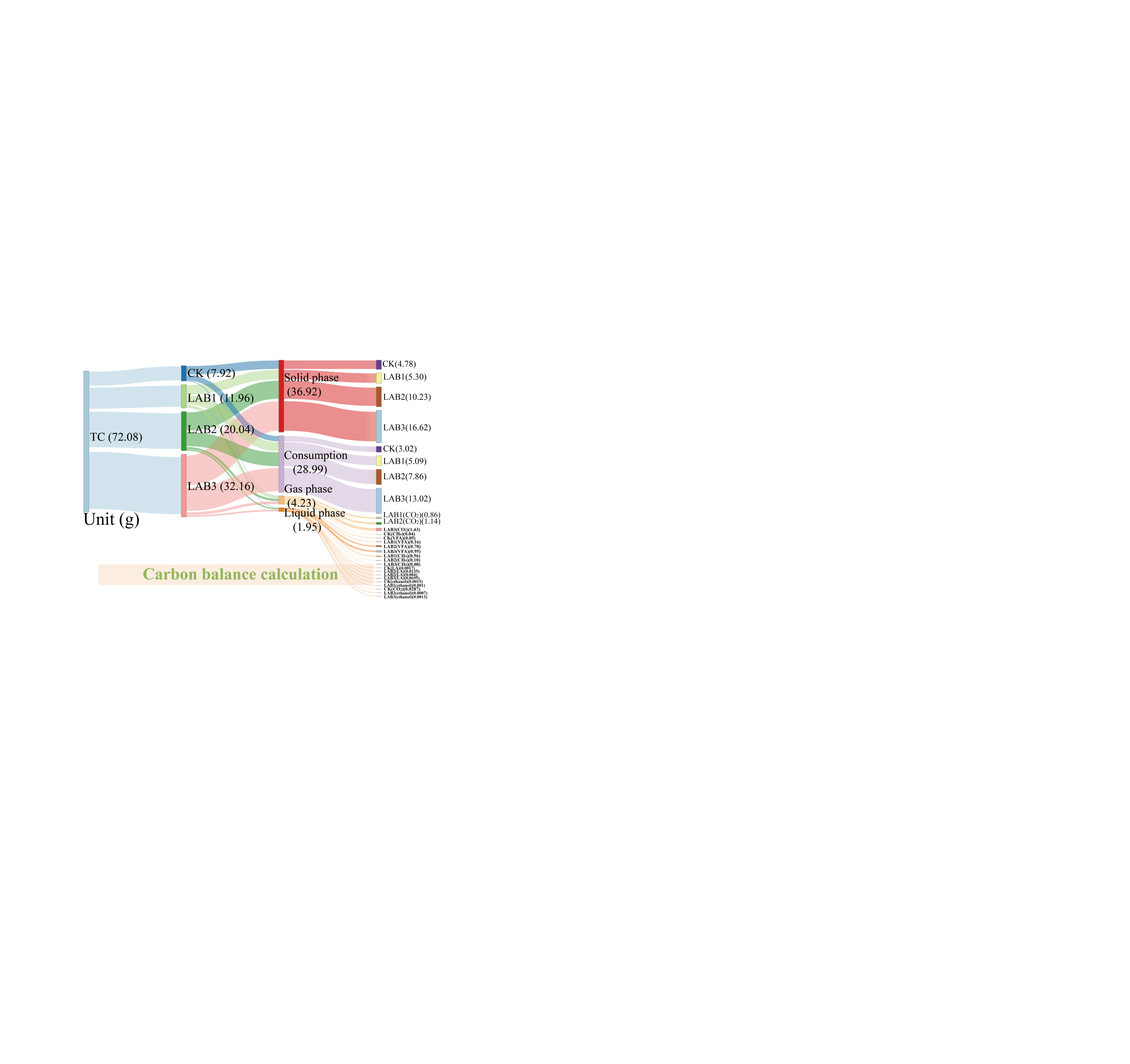


**Figure S2**. Carbon balance calculation under different LAB inoculation conditions.

**
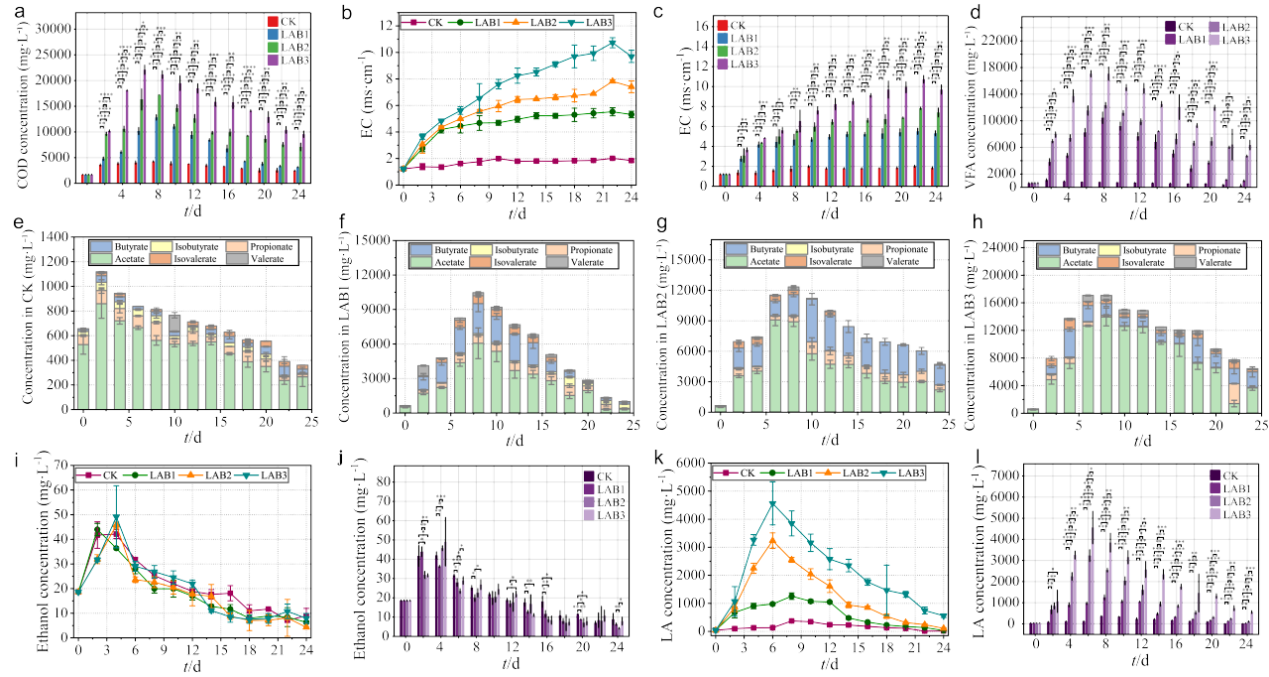
**

**Figure S3**. Visualization of significant tests for COD concentration (a), fluctuation in EC values (b), visualization of significant tests for EC values (c), visualization of significant tests for VFA concentration (d), fluctuation characteristics of different VFA components (e-h), fluctuation in ethanol concentration (i), visualization of significant tests for ethanol concentration (j), fluctuation in lactic acid concentration (k), visualization of significant tests for lactic acid concentration (l) under different LAB inoculation conditions. Significance was evaluated by *t*-test: **** P < 0.0001, *** P < 0.001, ** P < 0.01 and * P < 0.05.


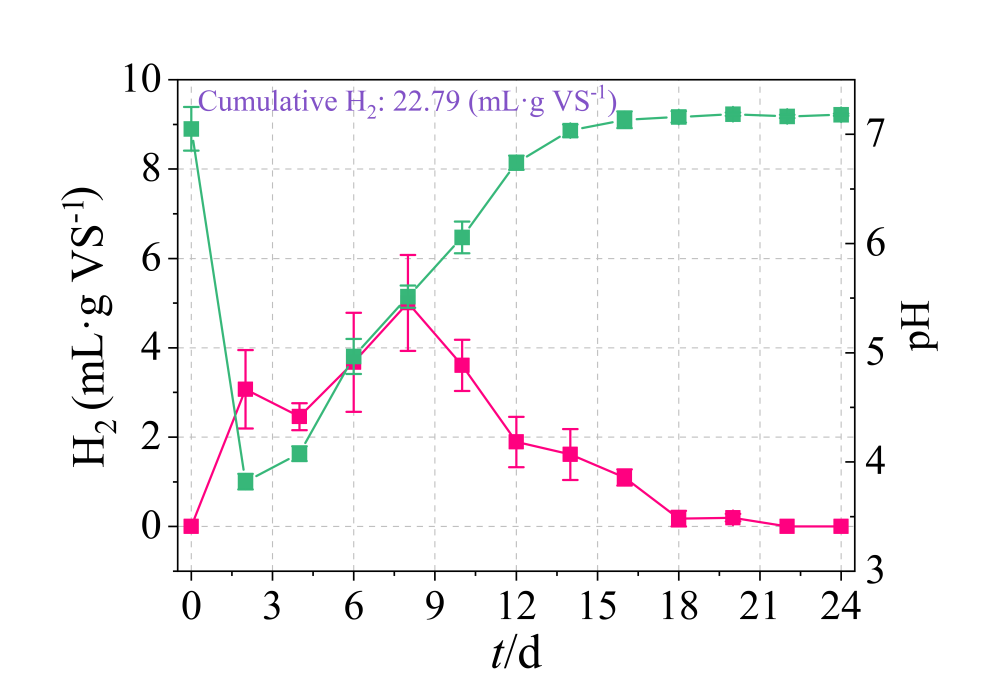


**Figure S4**. Fluctuations in daily H_2_ production (red dot) and pH value (green dot) in the hydrolysis verification test.

**
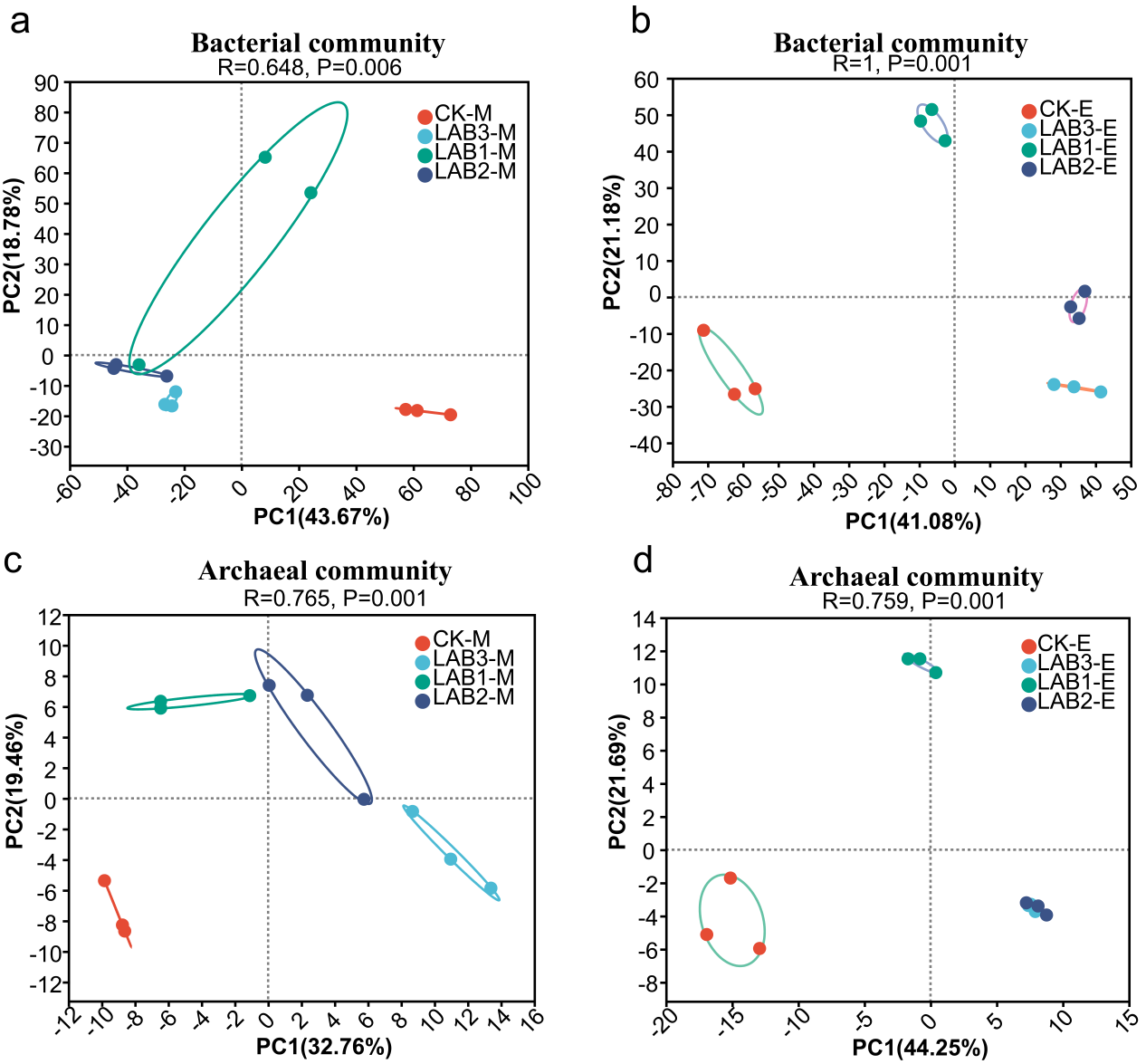
**

**Figure S5**. Principal component analysis of bacterial communities during first 6 days (a) and subsequent 18 days (b) under different LAB inoculation conditions. Principal component analysis of archaeal communities during first 6 days (c) and subsequent 18 days (d) under different LAB inoculation conditions.


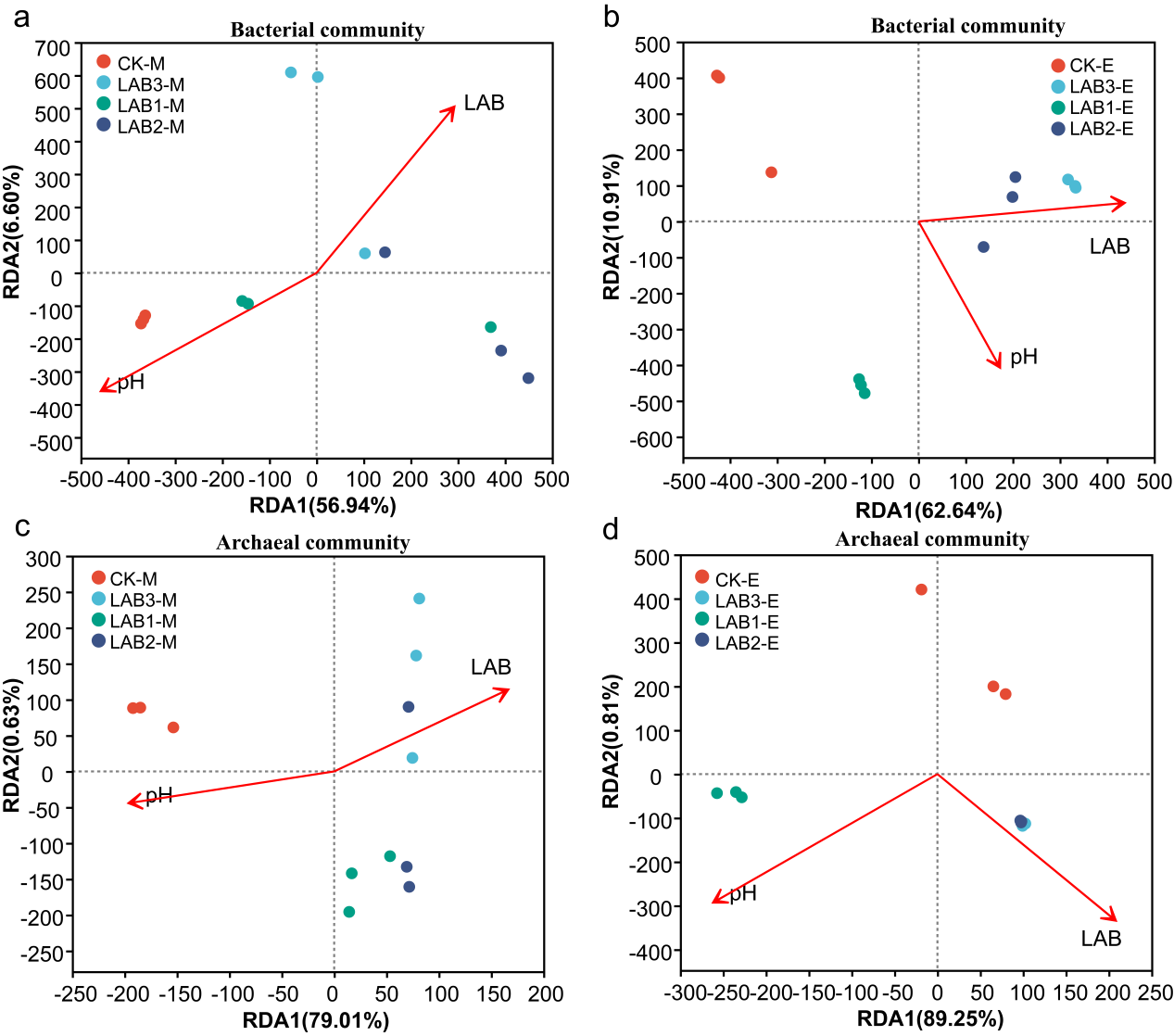


**Figure S6**. Redundancy analysis of bacterial communities during first 6 days (a) and subsequent 18 days (b) under different LAB inoculation conditions. Redundancy analysis of archaeal communities during first 6 days (c) and subsequent 18 days (d) under different LAB inoculation conditions. LAB and pH respectively represent two environmental factors.


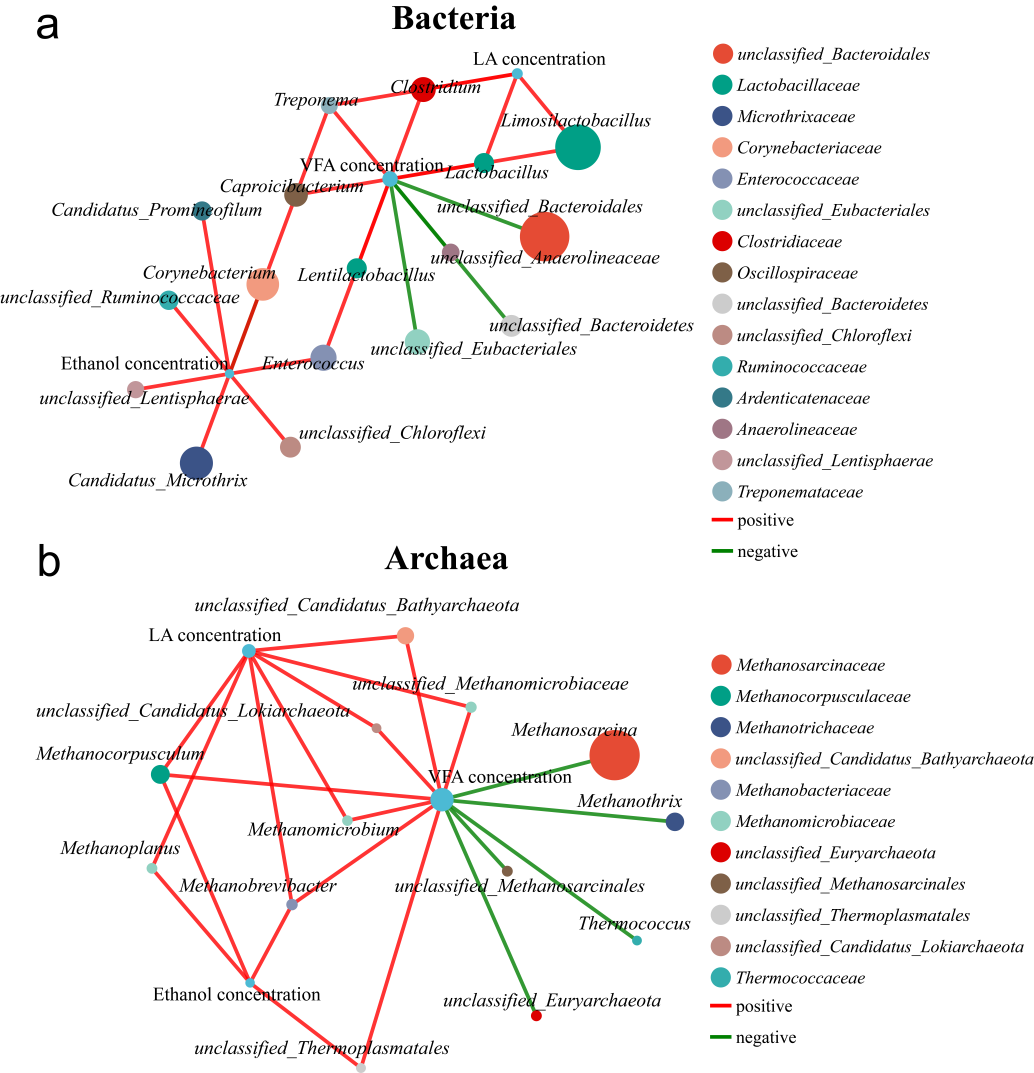


**Figure S7**. The correlation network based on *Pearson* correlation revealed a significant correlation (P < 0.05) between bacteria and metabolic products (VFA, lactic acid, ethanol) (a). The correlation network based on *Pearson* correlation demonstrated a significant correlation (P < 0.05) between archaea and metabolic products (VFA, lactic acid, ethanol) (b).


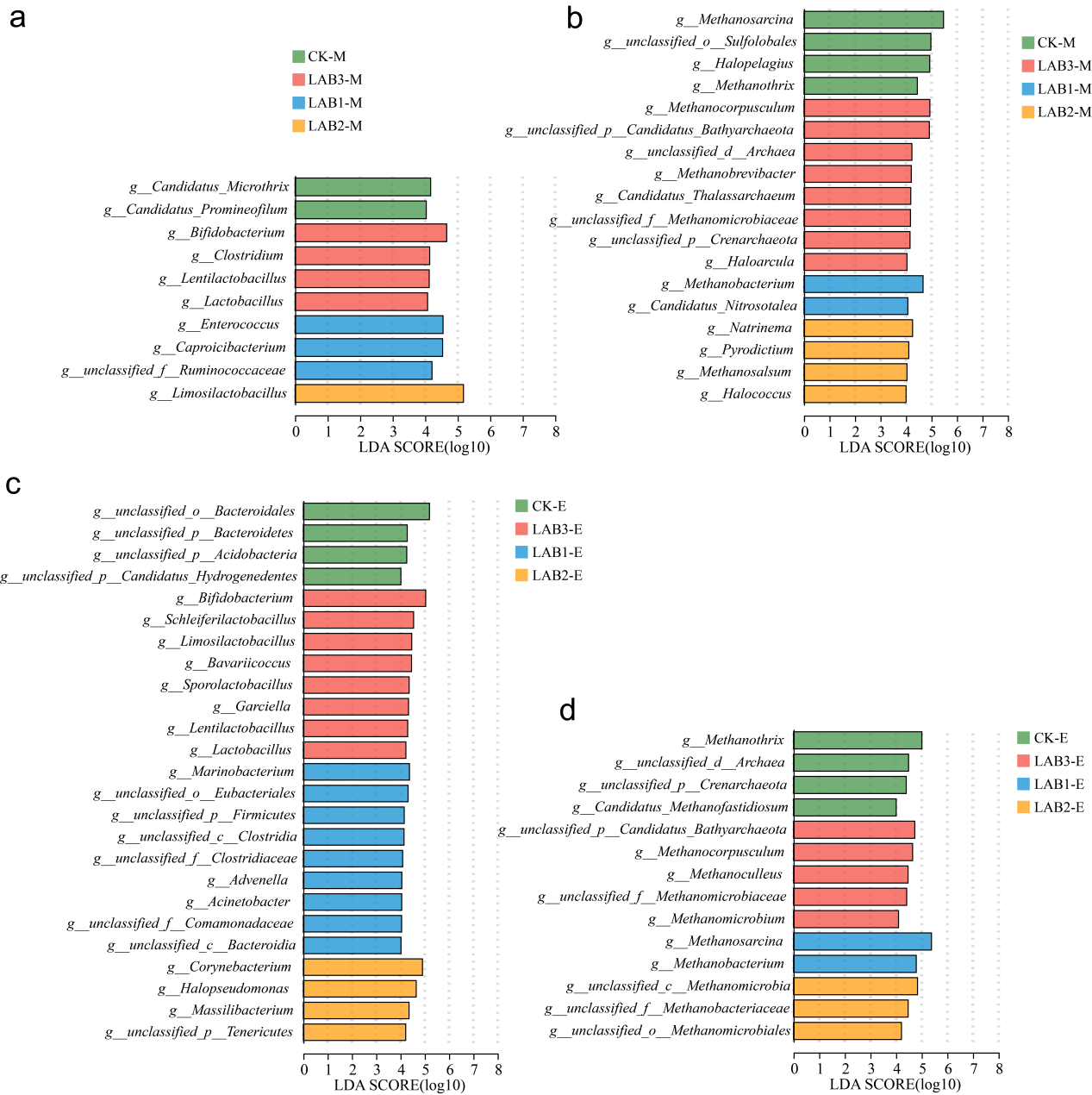


**Figure S8**. Linear discriminant analysis effect size (LEfSe) of bacteria (a) and archaea (b) during the first 6 days of AD in all systems, and LEfSe of bacteria (c) and archaea (d) in the subsequent 18 days of AD in all systems. LEfSe used linear discriminant analysis (LDA) to estimate the effect of the abundance of each genus on the difference of samples. LDA > 4 here.

**
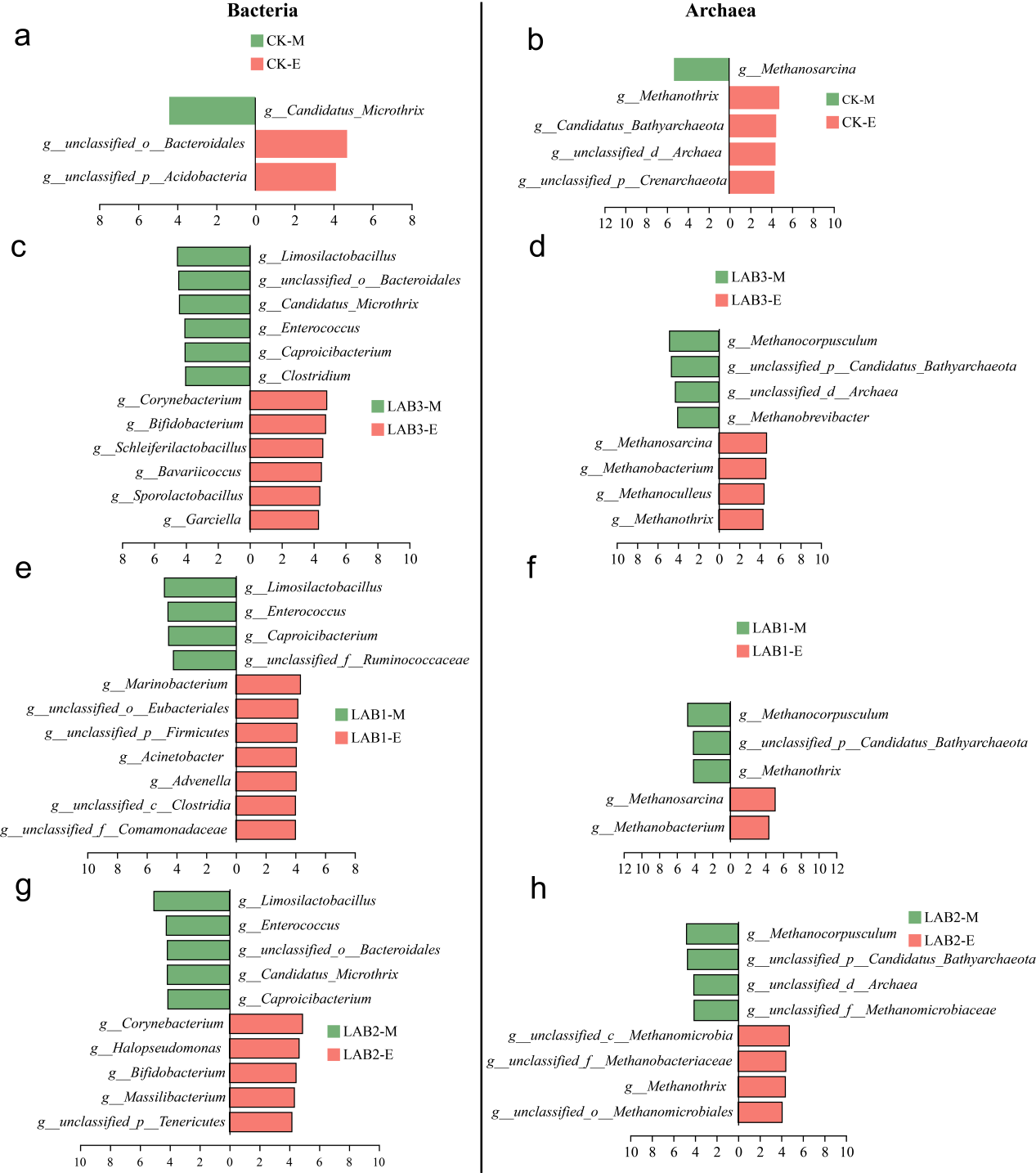
**

**Figure S9**. LEfSe of bacteria (a) and archaea (b) during the first 6 days and the subsequent 18 days of CK, LEfSe of bacteria (c) and archaea (d) during the first 6 days and the subsequent 18 days of LAB1, LEfSe of bacteria (e) and archaea (f) during the first 6 days and the subsequent 18 days of LAB2, LEfSe of bacteria (g) and archaea (h) during the first 6 days and the subsequent 18 days of LAB3. LEfSe used LDA to estimate the effect of the abundance of each genus on the difference of samples. LDA > 4 here.

**
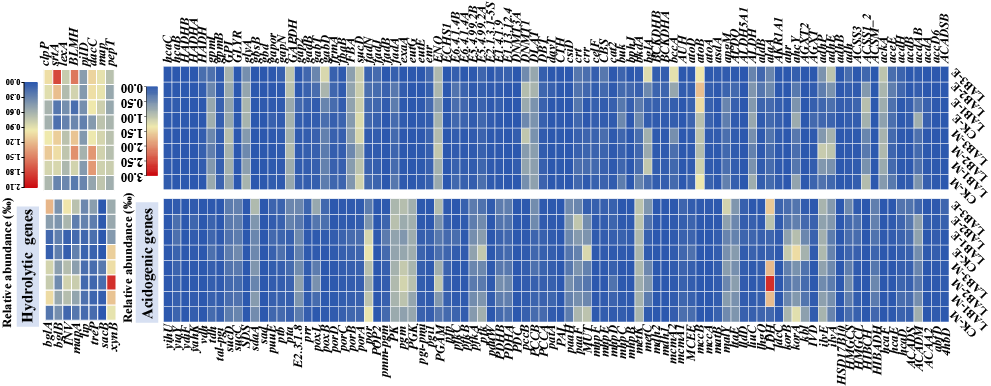
**

**Figure S10**. Relative abundance of genes involved in hydrolysis and VFA production.


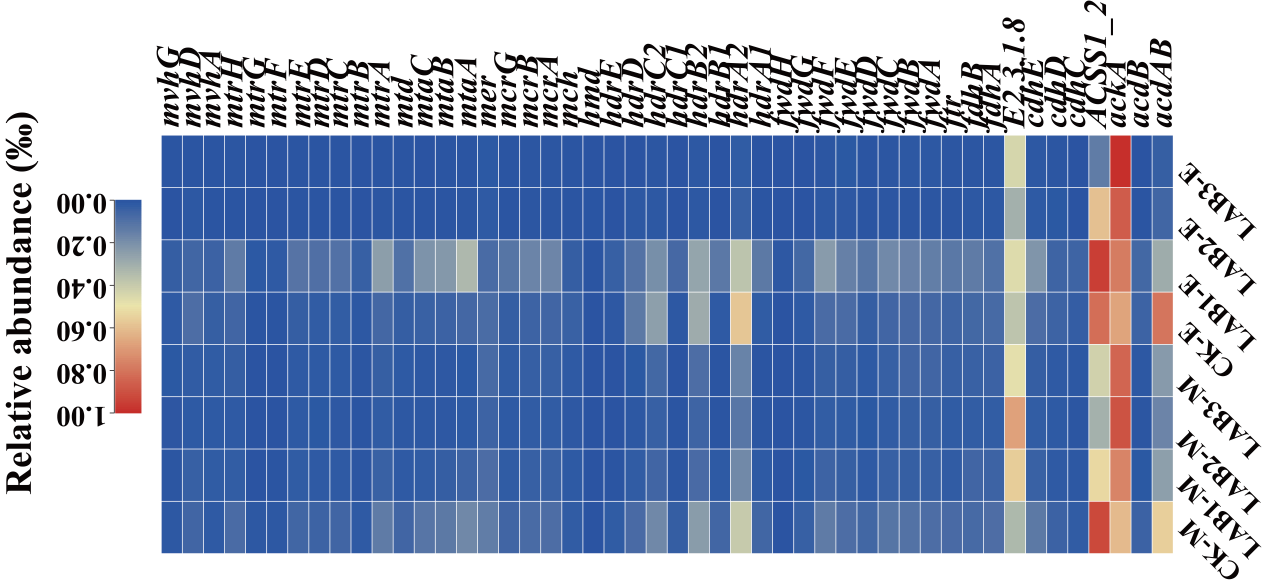


**Figure S11**. Relative abundance of genes involved in CH_4_ production.


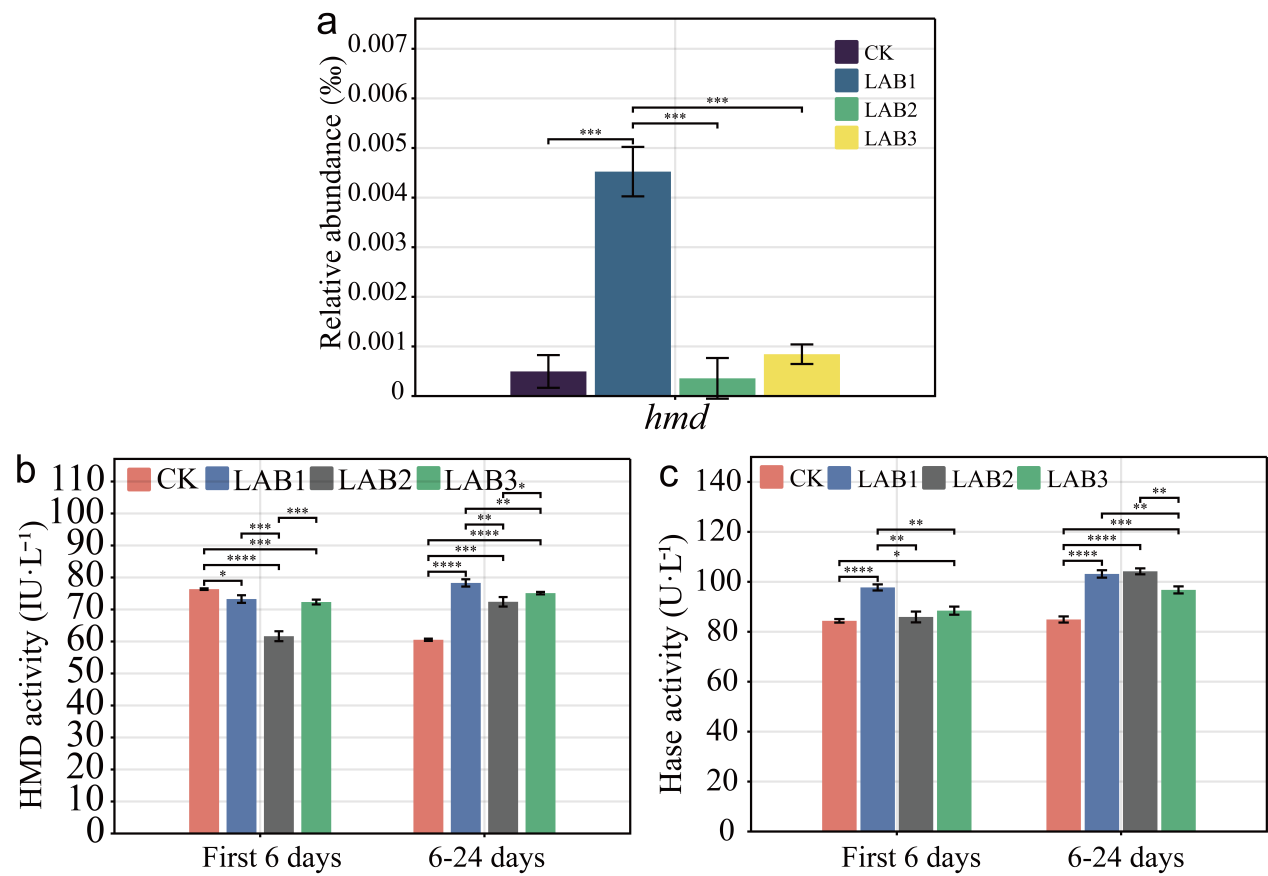


**Figure S12**. The relative abundance of *hmd* (a), HMD activity characteristics (b). Hase activity characteristics (c). Significance was evaluated by *t*-test: **** P < 0.0001, *** P < 0.001, ** P < 0.01 and * P < 0.05.

**Table S1**. Bacterial community alpha diversity under different LAB inoculation conditions

|  | chao | shannon | shannoneven | coverage |
| --- | --- | --- | --- | --- |
| CK-M | 4068.666667 | 0.078947333 | 0.508974 | 1 |
| CK-E | 4064.666667 | 0.155015667 | 0.446494667 | 1 |
| LAB1-M | 3988 | 0.073482333 | 0.491761 | 1 |
| LAB1-E | 3970.666667 | 0.026009333 | 0.567193667 | 1 |
| LAB2-M | 3929.666667 | 0.136187333 | 0.420533333 | 1 |
| LAB2-E | 3731.333333 | 0.070438 | 0.467880333 | 1 |
| LAB3-M | 3992 | 0.056763333 | 0.487696 | 1 |
| LAB3-E | 3516 | 0.106603 | 0.389968 | 1 |

**Table S2**. Archaeal community alpha diversity under different LAB inoculation conditions

|  | chao | shannon | shannoneven | coverage |
| --- | --- | --- | --- | --- |
| CK-M | 168 | 1.076614333 | 0.210132333 | 1 |
| CK-E | 176 | 2.154410333 | 0.416654333 | 1 |
| LAB1-M | 164 | 1.896422 | 0.371959333 | 1 |
| LAB1-E | 170 | 0.844034333 | 0.164347667 | 1 |
| LAB2-M | 162.6666667 | 2.407413667 | 0.472855667 | 1 |
| LAB2-E | 139 | 2.4274 | 0.491894333 | 1 |
| LAB3-M | 168 | 2.524486 | 0.492706667 | 1 |
| LAB3-E | 132.6666667 | 2.437277 | 0.498712333 | 1 |

Note: The index reflecting community richness is chao. The index that reflects the evenness of the community is shannoneven. The index reflecting community diversity is shannon. The index reflecting community coverage is coverage.

**Table S3** Molecular docking results of cellulose and *bglB*-encoding enzyme

| mode | affinity | dist from best mode | |
| --- | --- | --- | --- |
|  | (kcal/mol) | rmsd l.b. | rmsd u.b. |
| 1 | -5.9 | 0.000 | 0.000 |
| 2 | -5.7 | 27.488 | 30.248 |
| 3 | -5.2 | 26.818 | 30.089 |
| 4 | -5.0 | 27.027 | 30.346 |
| 5 | -5.0 | 28.286 | 31.539 |

**Table S4** Molecular docking results of hemicellulose and *xynB*-encoding enzyme

| mode | affinity | dist from best mode | |
| --- | --- | --- | --- |
|  | (kcal/mol) | rmsd l.b. | rmsd u.b. |
| 1 | -9.0 | 0.000 | 0.000 |
| 2 | -8.7 | 1.003 | 2.122 |
| 3 | -8.6 | 4.038 | 7.628 |
| 4 | -8.6 | 3.775 | 5.934 |
| 5 | -8.3 | 3.973 | 6.366 |

**References**

1. Kor-Bicakci, G., et al. Effect of dewatered sludge microwave pretreatment temperature and duration on net energy generation and biosolids quality from anaerobic digestion. ENERGY 168, 782-795 (2019).

2. Zhao, S.N. et al. Anaerobic co-digestion of chicken manure and cardboard waste: Focusing on methane production, microbial community analysis and energy evaluation. BIORESOURCE TECHNOLOGY 321 (2021).

3. Kang D D, et al. MetaBAT, an efficient tool for accurately reconstructing single genomes from complex microbial communities. PEERJ 3(8), 1165 (2015).

4. Sieber C M K, et al. Recovery of genomes from metagenomes via a dereplication, aggregation and scoring strategy. NATURE MICROBIOLOGY 3(7), 836 (2018).

5. Parks D H, et al. CheckM: assessing the quality of microbial genomes recovered from isolates, single cells, and metagenomes. GENOME RESEARCH 25(7), 1043 (2015).

6. Parks D H, et al. GTDB: an ongoing census of bacterial and archaeal diversity through a phylogenetically consistent, rank normalized and complete genome-based taxonomy. NUCLEIC ACIDS RESEARCH 50 (D1), D785-D794 (2022).
